# The association of ODF4 with AK1 and AK2 in mice is essential for fertility through its contribution to flagellar shape

**DOI:** 10.1038/s41598-023-28177-z

**Published:** 2023-02-20

**Authors:** Chizuru Ito, Tsukasa Makino, Tohru Mutoh, Masahide Kikkawa, Kiyotaka Toshimori

**Affiliations:** 1 Department of Functional Anatomy, Reproductive Biology and Medicine, Graduate School of Medicine, Chiba University, Chiba, 260-8670 Japan; 2 Department of Cell Biology and Anatomy, Graduate School of Medicine, The University of Tokyo, Tokyo, Japan; 3 Future Medicine Research Center, Chiba University, Chiba, 260-8670 Japan

**Keywords:** Cell biology, Infertility

## Abstract

Normal sperm flagellar shape and movement are essential for fertilization. The integral protein outer dense fiber 4 (ODF4) localizes to ODFs, but its function remains unclear. Adenylate kinase (AK) is a phosphotransferase that catalyzes the interconversion and controls the concentration equilibrium of adenine nucleotides. AK shuttles ATP to energy-consuming sites. Here, we report on the relationship of flagellar shape and movement with ODF4, AK1 and AK2 by using *Odf4*-deletion (*Odf4*^−/−^) mice. Soluble ODF4 is coimmunoprecipitated with AK1 and AK2 in *Odf4*^+/+^ spermatozoa. ODF4, AK1 and AK2 localize to whole flagella (plasmalemma, mitochondria, ODFs, and residual cytoplasmic droplets (CDs)), principal pieces, and midpieces, respectively. *Odf4*^−/−^ sperm flagella lose ODF4 and reduce AK1 and AK2 but produce ATP. The flagellum is bent (hairpin flagellum) with a large CD in the midpiece. There is no motility in the midpiece, but the principal piece is motile. *Odf4*^−/−^ spermatozoa progress backward and fail to ascend in the uterus. Thus, *Odf4*^−/−^ males are infertile owing to abnormal flagellar shape and movement caused mainly by the loss of ODF4 with AK1 and AK2. This study is supported by the rescue experiment; the abnormalities and male infertility caused by *Odf4* deletion were reversed by *Odf4* restoration.

## Introduction

Sperm energy is produced by the tricarboxylic acid (TCA) cycle in the midpiece (proximal flagellum) and by glycolysis in the principal piece (distal flagellum)^1^ (Fig. 2C, Fig. S2A). The energy produced is consumed for flagellar shape and movement. The normal flagellum has a straight shape. A straight flagellum is critical for symmetrical forward progression; flagellar shape defects and movement failure result in male infertility (World Health Organization: WHO)^2^. Flagellar shape and movement are supported by internal cytoskeletal proteins of the outer dense fibers (ODFs), fibrous sheath and axonemal microtubules^1^. ODFs (numbers 1–9) emanate from the axoneme in the neck and are concentrically arranged in the whole flagella. They are covered by helically arranged mitochondria in the midpiece and by a fibrous sheath in the principal piece and run down the center of the flagellum, surrounding the “9 + 2” microtubular axonemes. ODF4 is one of the main elements composing the ODFs. Mouse ODF4 is composed of 290 amino acids (approximately 33 kDa) encoded by the *Odf4* gene (4 exons, chromosome 11)^3^ (Mouse Genome Informatics: MGI). Human ODF4 (also known as cancer/testis antigen 136) is composed of 257 amino acids (30 kDa: 5 exons, 17p13.1); the deduced 257-amino acid human ODF4 protein shares 39.9% identity with its mouse protein counterpart^4^ (National Center for Biotechnology Information: NCBI). ODF4 was initially called OPPO1 (a Japanese word for tail) and thought to be one of the sperm cytoskeletal proteins^3^, but the function of ODF4 remains unclear^1^ (NCBI). We have termed such a hardly soluble form of ODF4 localized in the ODFs an ‘integral ODF4’ in this report.

A bent flagellum is one of the most common abnormalities in sperm shape and movement in infertile male patients^2^. A bent flagellum with an angle of 180 degrees is called a hairpin sperm flagellum (hairpin flagellum for short) and moves asymmetrically. The hairpin flagella have a large residual cytoplasm in the midpiece. Under normal conditions, most mature spermatozoa that reach the cauda epididymis have little residual cytoplasm in the flagellum, referred to as cytoplasmic droplets (CDs)^5,6^; CDs are found in vertebrate species from fish to humans^7^. During mammalian sperm maturation in the epididymis, a CD is formed around the base of the head at the end of spermatogenesis and moves toward the annulus, a circular structure or cortex ring that functions as a junction between the midpiece and principal piece; eventually, the CD is found as a tiny membrane-bound vesicle in the cauda sperm flagella^1^ (Fig. S2A, Fig. 8). A CD is generally shed from a spermatozoon in the distal epididymis or in the uterus soon after copulation, and then flagellar straightening occurs. Therefore, CD shedding has long been thought to be physiological. However, the mechanisms of how flagellar shape and movement are related to energy production and consumption remains unclear.

Adenine nucleotides (AMP, ADP and ATP) are essential for cellular life, including for ciliary movement and metabolism^8^, and any defect in them causes severe primary ciliary dyskinesia in humans^9^. Regarding sperm energy production, there are reports showing that most of the energy for flagellar motility is produced by glycolysis through a glycolytic enzyme, the sperm-specific isoform of glyceraldehyde 3-phosphate dehydrogenase (GAPDS), in the fibrous sheath rather than by mitochondrial oxidative phosphorylation in the midpiece^10,11^. The produced ATP is consumed by cytosolic and axial dynein ATPases^10–12^. Adenylate kinase (AK, also known as myokinase: EC 2.7.4.3) is a phosphotransferase ubiquitously distributed throughout the body that catalyzes the interconversion of adenine nucleotides. AK shuttles ATP to energy-consuming sites^8^. AK recruits two molecules of ADP and reversibly transfers phosphate groups to one molecule each of ATP and AMP (2 ADP ⇔ ATP + AMP), where the equilibrium constant is close to 1^13,14^. Thus, AK controls the concentration equilibrium of adenine nucleotides to balance energy production and consumption. To date, three adenylate kinases have been reported in spermatozoa: AK1, AK2^13,15,16^ and AK8^17^. AK1 (23 kDa) localizes to ODFs^15^. AK2 (30 kDa) localizes to the mitochondria^15^. AK8 (56 kDa) localizes to the axoneme^17^. One line of *AK1* knockout (KO) mice produced spermatozoa with no remarkable effect on flagellar motility^16^. The other lines of *AK1* KO mice impaired cardioprotective signaling^18^, blunted the vascular adenylate kinase phosphotransfer system, failed adequate coronary reflow following ischemia‒reperfusion^19^, and impaired skeletal myocyte energy homeostasis^20^. There are no *AK2* KO or *AK8* KO mice.

Recently, the role of ODF-related proteins has become recognized as an important issue in disease. Our previous contribution to this topic indicated that the cytoskeletal protein ODF2 plays a role in fertility because *Odf2*-haploinsufficient males were infertile owing to unique sperm head–tail separation^21^. In this study, we report the contribution of ODF4 to fertility, where ODF4 associates with AK1 and AK2 to control energy metabolism for sperm shape (flagellar straightening) and movement.

## Results

### *Odf4* expression and localization of ODF4, AK1 and AK2

**Figure 1:**
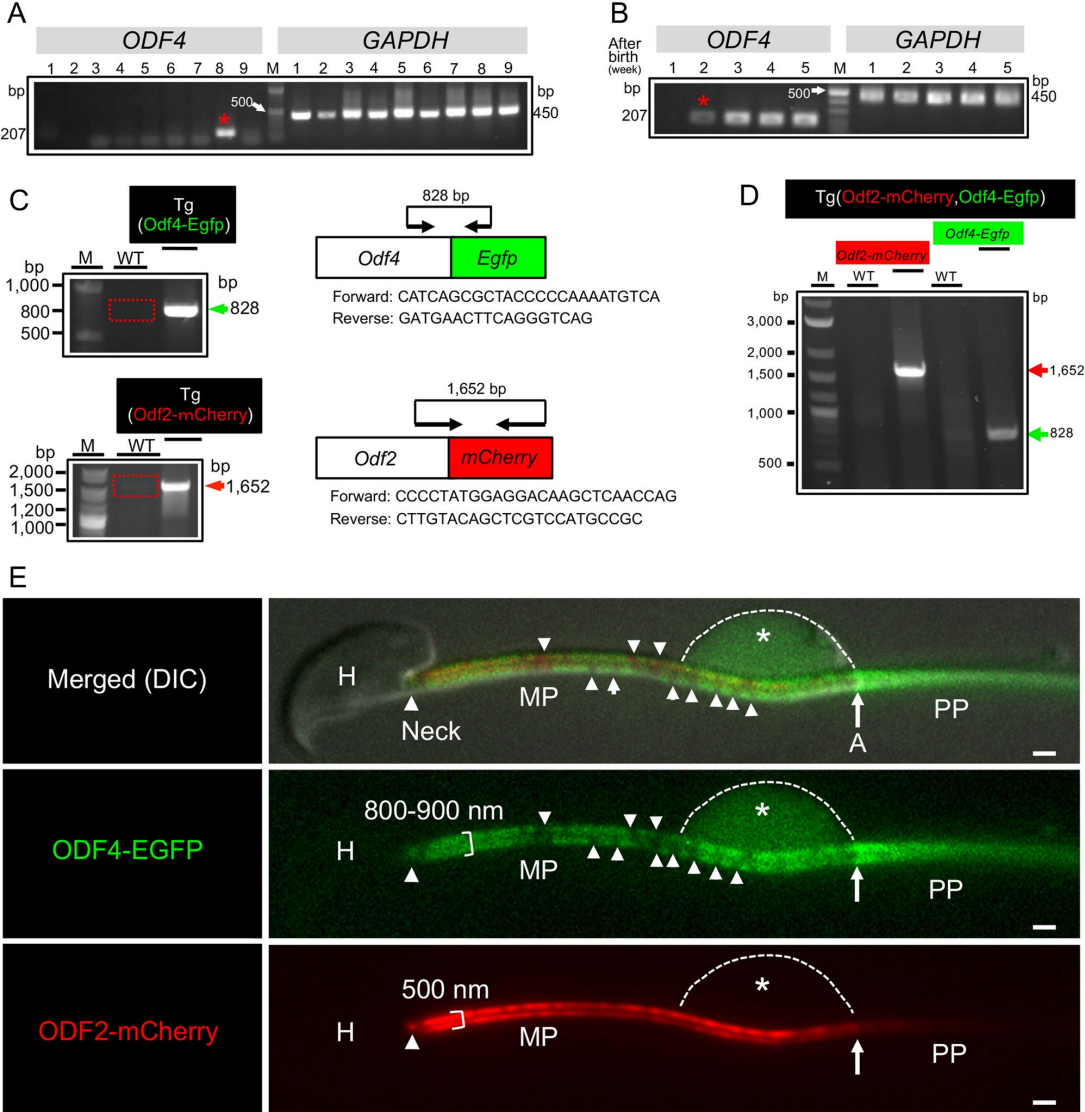
*Odf4* and ODF4 expression, Tg generation, and localization difference between ODF4 and ODF2. (**A–D**) Cropped blots from the same gel. Original full-length images are shown in the supplementary Fig S1. (**A**,**B**) RT‒PCR. (**C**,**D**) Genomic PCR. (**A**) *ODF4*: 207 base pairs (bp). *GAPDH*: 450 bp (Control). M, Marker proteins. *ODF4* is expressed only in the testis (_*_: lane 8). 1, Brain: 2, Heart: 3, Lung: 4, Liver: 5, Spleen: 6, Kidney: 7, Small intestine: 8, Testis: 9, Epididymis. (**B**) *ODF4* is expressed first at 2 weeks (_*_) after birth. (**C**) (Top) *Odf4-Egfp* (828 base pairs (bp) and primers) contains inserted Tg (arrowhead) but not in the wild-type (WT: square). (Bottom) Generation of Tg(Odf2-mCherry)*. Odf2-mCherry* (1,652 bp and primers) was inserted in Tg (arrowhead) but not in the wild-type (WT: square). (**D**) Generation of double Tg(Odf2-mCherry,Odf4-Egfp). Green and red arrowheads indicate the inserted *Odf4-Egfp* (828 bp) and *Odf2-mCherry* (1,652 bp), respectively. (**E**) Localization comparison between ODF4 and ODF2. Fluorescence signals in double Tg(Odf2-mCherry,Odf4-Egfp) cauda spermatozoa. Merged (DIC): DIC image of merged ODF2-mCherry (red) and ODF4-GFP (green) signals. The width of the ODF4-EGFP green signal (800–900 nm) is larger than that of ODF2-mCherry (approximately 500 nm). The green signal is observed as a rather striped pattern in the midpiece reflecting mitochondrial arrangement (arrowheads), whereas it is linearly seen in the principal piece (PP). A green signal is observed in an expanded CD (*). Scale bar = 1 μm.

*ODF4* was expressed in the testis (Fig. 1A, Fig. S1A). It was first observed in the second week after birth (Fig. 1B, Fig. S1B), when spermatocytes first appeared. To clarify the function of ODF4 and to localize ODF4, we established the Tg(Odf4-Egfp) and double Tg(Odf2-mCherry,Odf4-Egfp) mouse lines (Fig. 1C, D and Figs. S1C, D, respectively). The width of the ODF4-EGFP green signal (800–900 nm) was larger than that of the ODF2-mCherry red signal (approximately 500 nm) of another cytoskeletal protein (Fig. 1E), suggesting some role(s) in addition to its cytoskeletal nature. The ODF4-EGFP signal was detected in the entire flagella of postcopulatory spermatozoa recovered from the uterine lumen (Fig. S2B). In cauda epididymal sperm, AK1 was mainly detected in the principal piece (Figs S2D, E and S3) and in the CD in the distal midpiece near the annulus (Figs S2C–E). In contrast, AK2 was mainly detected in the midpiece (Figs S2D, E and S3) and in the CD near the annulus (Figs S2C, D and S3) of cauda epididymal sperm. AK1 and AK2 were hardly detected in the *Odf4*^−/−^ spermatozoa (Fig. S3), and their reduced expression was also shown later by western blotting (Fig. 4, Fig. S6). These data are summarized in a table (Fig. S2E). Immunoelectron microscopy using an antibody against GFP for Tg(Odf4-Egfp) spermatozoa revealed that immunogold particles labeled ODF4-EGFP were localized to the plasmalemma, mitochondria and ODFs along the full length of the flagellum, but the CD disappeared due to repeated washings and centrifugations during sample preparation (Fig. S4).

### Characterization of *Odf4*^−/−^ mice and spermatozoa

**Figure 2:**
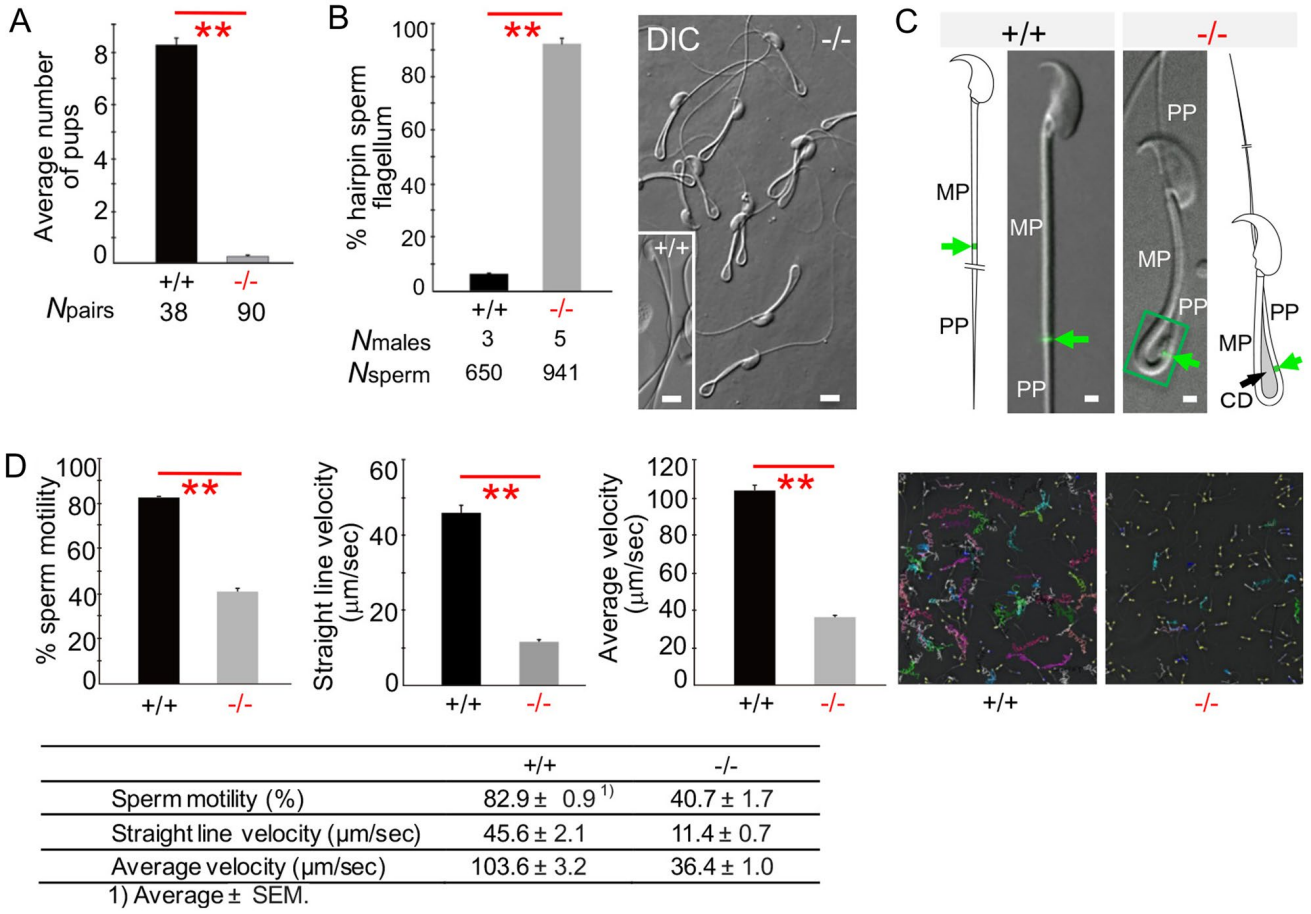
Phenotype of *Odf4*^−/−^ males and spermatozoa. + / + : *Odf4*^+/+^. −/−: *Odf4*^−/−^. (**A**) *Odf4*^−/−^ males are infertile. Average number of pups (fertility rate): 8.2 ± 0.3 (+ / +) versus 0.3 ± 0.1 (−/−). Data are presented as the average ± standard error of the mean. The *p value* was calculated using the Mann‒Whitney U test (two-tailed analysis) using the add-in Statcel 3 software (OMS) for Microsoft Excel. (**B**) Postcopulatory hairpin sperm flagella. (Left) Percentage (%) of hairpin sperm flagella: 6.3 ± 0.8 (+ / +) versus 92.3 ± 1.9. Student’s t test (two-tailed analysis). (Right) DIC images. Inset: + / + . Scale bar = 5 μm. (**C**) Cytoplasmic droplet (CD) shown by immunofluorescence with an antibody against SEPTIN 7 (green signal: arrows) for *Odf4*^−/−^ and *Odf4*^+/+^ cauda epididymal spermatozoa. CD is absent, but SEPTIN 7 is present at the annulus region in *Odf4*^+/+^. In contrast, both CD and SEPTIN 7 are present in the annulus region in *Odf4*^−/−^ mice. The internal component of the *Odf4*^−/−^ indicated at a rectangular area is further shown by TEM (Fig. 3D). MP: midpiece. PP: principal piece. Scale bar = 1 μm. (**D**) SMAS and the raw data showing the reduction in *Odf4*^−/−^ sperm movement. (Left) Percentage (%) sperm motility. (Middle left) Straight line velocity. (Middle right) Average velocity. All of these factors were significantly reduced in *Odf4*^−/−^ mice compared to *Odf4*^+/+^ mice. Welch’s t test (two-tailed analysis). (Right) Representative images of SMAS before capacitation. (Bottom) The table shows raw data for SMAS. *Odf4*^−/−^ results in poor motility, where each color shows an individual spermatozoon. The total number of males (spermatozoa) examined was 3 (7,009) and 6 (14,911) for *Odf4*^−/−^ and *Odf4*^+/+^ males, respectively. **P < 0.01.

**Figure 3:**
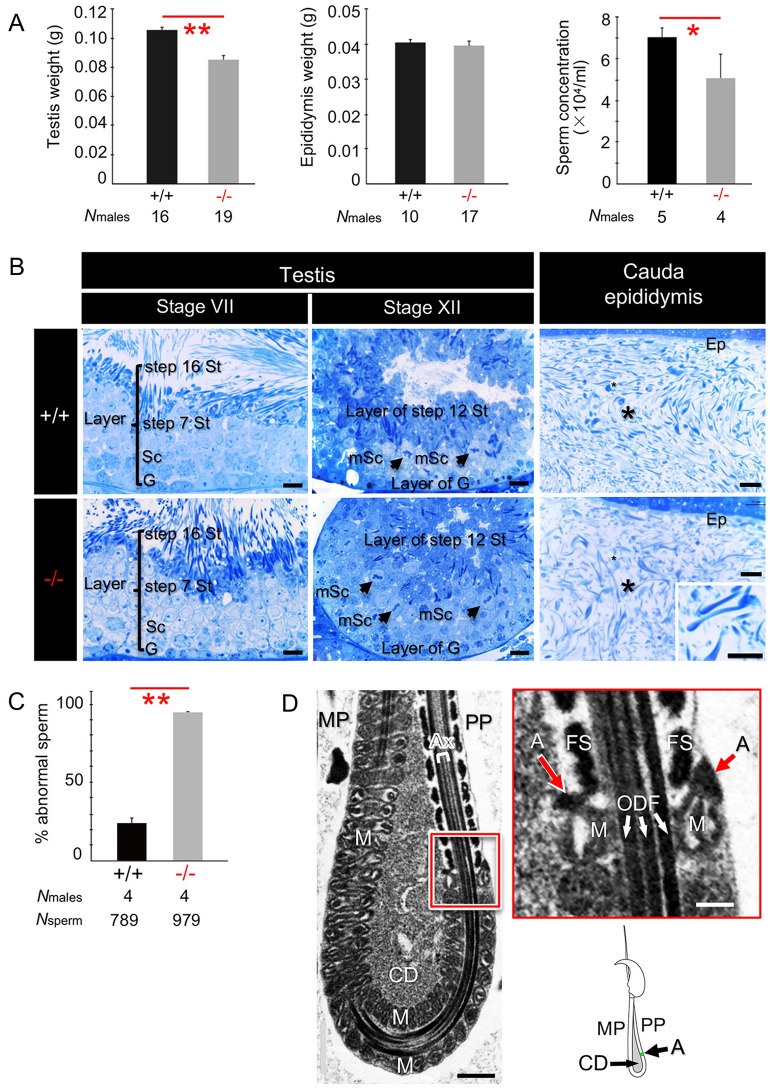
Characterization of *Odf4*^−/−^ males. + / + : *Odf4*^+/+^. −/−: *Odf4*^−/−^. (**A**) Reproductive organs. (Left) Testis weight (g): 0.11 ± 0.00 (+ / +) versus 0.09 ± 0.00 (−/−). Student’s t test (two-tailed analysis). (Middle) Epididymis weight (g): 0.04 ± 0.00 (+ / +) versus 0.04 ± 0.00 (−/−). (Right) Sperm concentration (× 10^4^/mL): 7.1 ± 0.5 (+ / +) versus 5.1 ± 1.2 (−/−). Mann‒Whitney U test (two-tailed analysis). (**B**) Spermatogenesis (testis) and spermatozoa in the cauda epididymal lumen (cauda epididymis). Toluidine blue-stained light micrographs. (Left and Middle) Normal spermatogenesis, where developing spermatogenic cells and layers at stages VII (Left) and XII (Middle) are observed. (Right) The cauda epididymal lumen contains a large number of spermatozoa (*) in both *Odf4*^+/+^ and *Odf4*^−/−^ mice. (Inset) in *Odf4*^−/−^; hairpin flagella are observed. Ep: Epithelia. G: Spermatogonia. Sc: Spermatocytes. mSc: spermatocytes during meiosis. St: Spermatids. N = 3 different males. Scale bar = 20 μm for stages VII and XII and 10 μm for the cauda epididymis. (**C**) Percent (%) abnormal spermatozoa. 24.2 ± 3.1 (+ / +) versus 94.5 ± 0.3 (−/−). Welch’s t test (two-tailed analysis). (**D**) TEM showing normal organization of flagellar internal components in *Odf4*^−/−^ flagella. (Left) Presence of a cytoplasmic droplet (CD) shown by longitudinal section of the hairpin region. (Top, Right) High magnification of the rectangular area of the left image, with a schematic drawing of hairpin flagellum (Bottom). *A* Annulus, *Ax* Axial filaments (not clearly found at this magnification). *FS* Fibrous sheath, *M* Mitochondria, *MP* Midpiece, *ODF* Outer dense fibers, *PP* Principal piece. *P < 0.05. **P < 0.01. Scale bar = 0.5 μm, 100 nm for inset (red line, square).

**Figure 4:**
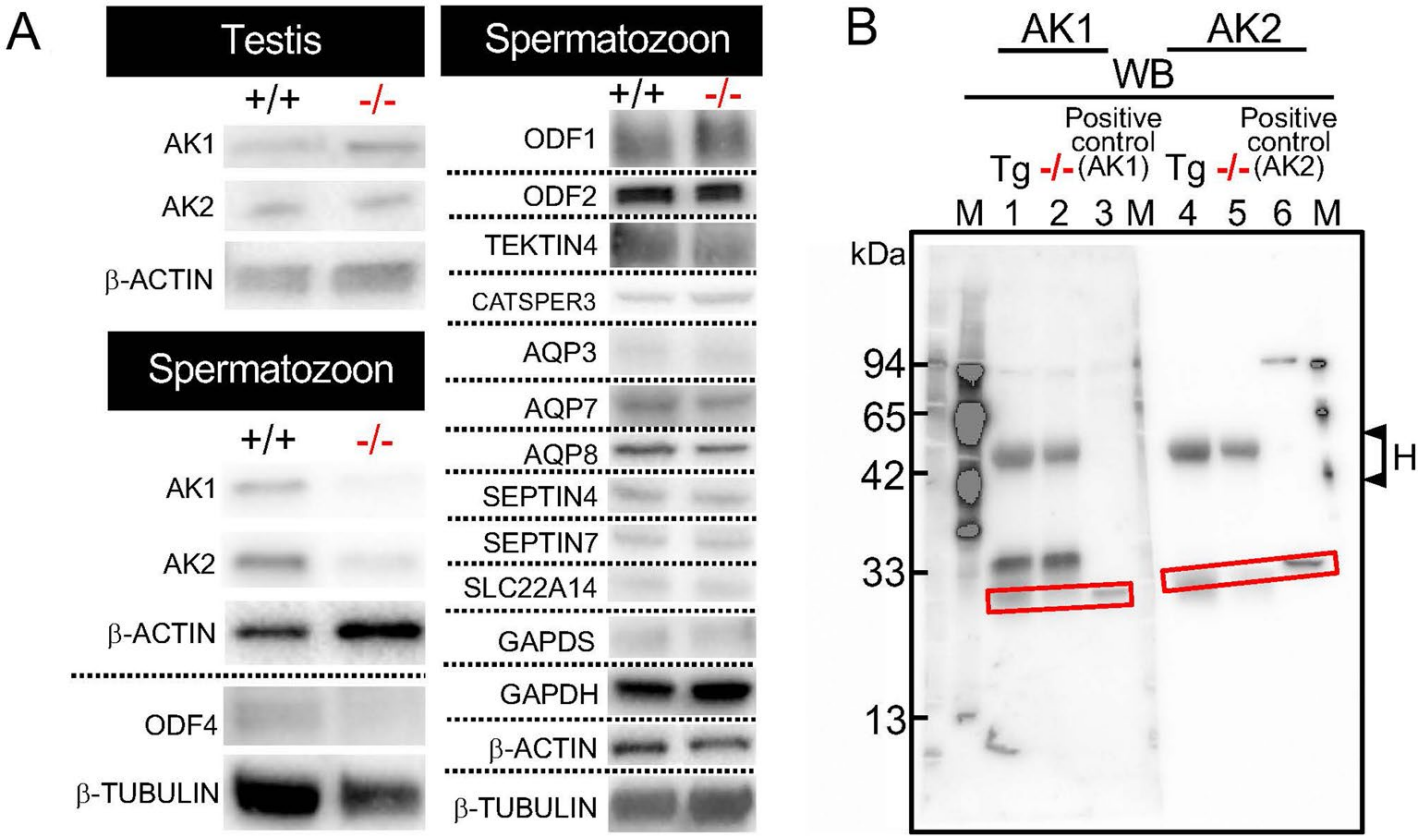
AK1 and AK2 in testis, spermatozoa, flagellar proteins (**A**) and immunoprecipitation (IP) (**B**)**.** + / + : *Odf4*^+/+^. −/−: *Odf4*^−/−^. Western blotting. (**A**,**B**) Cropped images. (**A**) (Left) Testicular and cauda epididymal sperm extracted samples. β-ACTIN and β-TUBULIN: internal controls. AK1 and AK2 are detected in both *Odf4*^+/+^ testes (Top) and cauda spermatozoa (Bottom); however, they are detected in *Odf4*^+/+^, but reduced or hardly detected in *Odf4*^−/−^ cauda spermatozoa (Bottom). Further cropped images are shown in the supplementary Fig S6 for testis (Top) and spermatozoon (Bottom). These original full-length images with different exposure times are shown in the supplementary SD2 for testis (Top) and SD3 spermatozoon (Bottom). (Right) All flagellar proteins extracted from *Odf4*^+/+^ and *Odf4*^−/−^ mice examined are immunopositive for each corresponding antibody: ODF1, ODF2, TEKTIN4, CATSPER3, AQP3, AQP7, AQP8, SEPTIN4, SEPTIN7, SLC22A14, GAPDS, GAPDH, β-ACTIN and β-TUBULIN. Further cropped images are shown in the supplementary Fig S7. These original full-length images with different exposure times are shown in each corresponding supplementary SD from SD4 to SD13. (**B**) Cropped images of IP with anti-GFP antibody for the extracts in RIPA buffer from Tg(Odf4-Egfp) and *Odf4*^−/−^ (control) spermatozoa and western blotting with anti AK1 (23 kDa) and AK2 (30 kDa) antibodies for the same blotted membrane after SDS-PAGE. Lanes 1 and 4 (Tg): Tg(Odf4-Egfp) sperm extracts. Lanes 2 and 5: *Odf4*^−/−^ (−/−) sperm extracts. Lanes 3 and 6: Positive control (IP-untreated *Odf4*^+/+^ samples) to identify AK1 and AK2, respectively. The AK1 and AK2 signals are clearly detected in lanes 1 and 4 for Tg and lanes 3 and 6 for the positive control, but they are not found in lanes 2 and 5 for *Odf4*^−/−^ (−/−), respectively (rectangles). These original full-length images with different exposure times are shown in the supplementary SD14. H on the right side indicates the position of the IgG heavy chain (approximately 55 kDa). M, marker proteins (protein molecular marker and/or prestained marker).

To investigate the physiological role of ODF4 in vivo, we deleted the *Odf4* gene. Ablation at the desired region was confirmed by DNA sequence analysis (Fig. S5A) and genomic PCR analysis (Fig. S5B). Western blotting and immunofluorescence assays detected no ODF4 signals in the *Odf4*^−/−^ samples (Figs S5C, D). Nonspecific genome modifications were not observed in at least two potential off-target sequences. *Odf4*^−/−^ females were healthy and exhibited no overt abnormalities. *Odf4*^−/−^ males were healthy and normal vaginal plugs formed when they mated with females; however, they were almost completely infertile, which resulted in a severely reduced average number of pups (Fig. 2A). The *Odf4*^−/−^ testis weight was significantly reduced, but the epididymis weight was not significantly different between *Odf4*^+/+^ and *Odf4*^−/−^ mice (Fig. 3A). The cauda epididymal sperm concentration was significantly reduced in *Odf4*^−/−^ mice compared to *Odf4*^+/+^ mice (Fig. 3A). *Odf4*^−/−^ males exhibited all stages of germ cell differentiation from spermatogonia to spermatozoa in the seminiferous epithelium and produced normal-looking spermatozoa (Fig. 3B). However, mature spermatozoa that had reached the cauda epididymis displayed a high percentage (more than 90%) of morphological abnormalities (Fig. 3C), including hairpin-shaped flagella (Fig. 2B). The annulus architecture appeared normal, as confirmed by transmission electron microscopy (TEM) (Fig. 3D). The annulus-specific marker SEPTIN 7 was normally detected in the annulus region, as verified by both immunofluorescence (Fig. 2C) and western blotting (Fig. 4A and Fig. S7). However, the CD was retained around the annulus region, spanning from the distal midpiece to around the annulus region, whereas all other flagellar internal components, such as ODFs, fibrous sheath and axial filaments, appeared normal, as shown by TEM (Fig. 3D). A computer-assisted sperm motility analysis system (SMAS) showed that all parameters, such as the percentage (%) of sperm motility, straight line velocity (μm/sec) and average velocity (μm/sec), were significantly reduced in *Odf4*^−/−^ spermatozoa compared to that of *Odf4*^+/+^ spermatozoa (Fig. 2D). Almost none of the *Odf4*^−/−^ spermatozoa were able to progress forward, instead progressing backward with immotile midpieces (Videos S1 and S2 for *Odf4*^+/+^ and *Odf4*^−/−^, respectively). This motility characteristic is further quantitatively analyzed below (Fig. 6).

### AK1 and AK2 in spermatozoa and immunoprecipitation (IP)

Next, we examined what kinds of flagellar proteins are present or absent in *Odf4*^−/−^ testicular and sperm cells using western blotting. For this purpose, to pay particular attention to the readily soluble form of ODF4 localized in the cytosol (here termed ‘soluble ODF4’), we collected the sample as described in detail in the Methods section; in short, we directly placed the samples without washing them into RIPA buffer containing 1% Nonidet P40. As a result, antibodies against AK1 and AK2 detected the corresponding signals in both control *Odf4*^+/+^ testis and cauda sperm extracts and in *Odf4*^−/−^ seminiferous tubules cell extracts, but these signals were significantly reduced in *Odf4*^−/−^ mature sperm cell extracts (Fig. 4A *Left* and Fig. S6). Signals for all other proteins examined were detected in both *Odf4*^−/−^ and *Odf4*^+/+^ cell extracts as follows: ODF1, ODF2, TEKTIN4, cation channel sperm associated 3 (CATSPER3), AQP3, AQP7, AQP8, SEPTIN 4, SEPTIN 7, solute carrier family 22 member 14 (SLC22A14), GAPDS, and GAPDH (Fig. 4A *Right* and Fig. S7). Next, we examined whether AK1 and AK2 can be coimmunoprecipitated with ODF4 in Tg(Odf4-Egfp) mice by IP using protein-G immunobeads conjugated with an antibody against GFP. For this purpose, samples were extracted from Tg(Odf4-Egfp)*Odf4*^+/+^ and *Odf4*^−/−^ spermatozoa (control). Both AK1 and AK2 proteins were coimmunoprecipitated with ODF4 (ODF4-EGFP) from the Tg(Odf4-Egfp)*Odf4*^+/+^ sperm extracts but not from the negative control *Odf4*^−/−^ sperm extract (Fig. 4B).

### Sperm ascension in the female reproductive tract

Because *Odf4*^−/−^ sperm motility was reduced, we investigated whether *Odf4*^−/−^ spermatozoa could ascend in the *Odf4*^+/+^ female reproductive tract after plug formation. For this purpose, we employed Tg(Eqtn-Egfp) male mice and females treated with hormones to control the estrous cycles for copulation. As a result, the control *Odf4*^+/+^ spermatozoa normally ascended in the uterus, entered the oviduct, reached the ampulla and fertilized eggs (Figs S8A–D). In contrast, *Odf4*^−/−^ spermatozoa stalled in the lower region of the uterine cavity, failed to ascend in the uterus and were unable to fertilize eggs (Figs S8E–H).

### ATP and ADP concentrations before and after capacitation

**Figure 5:**
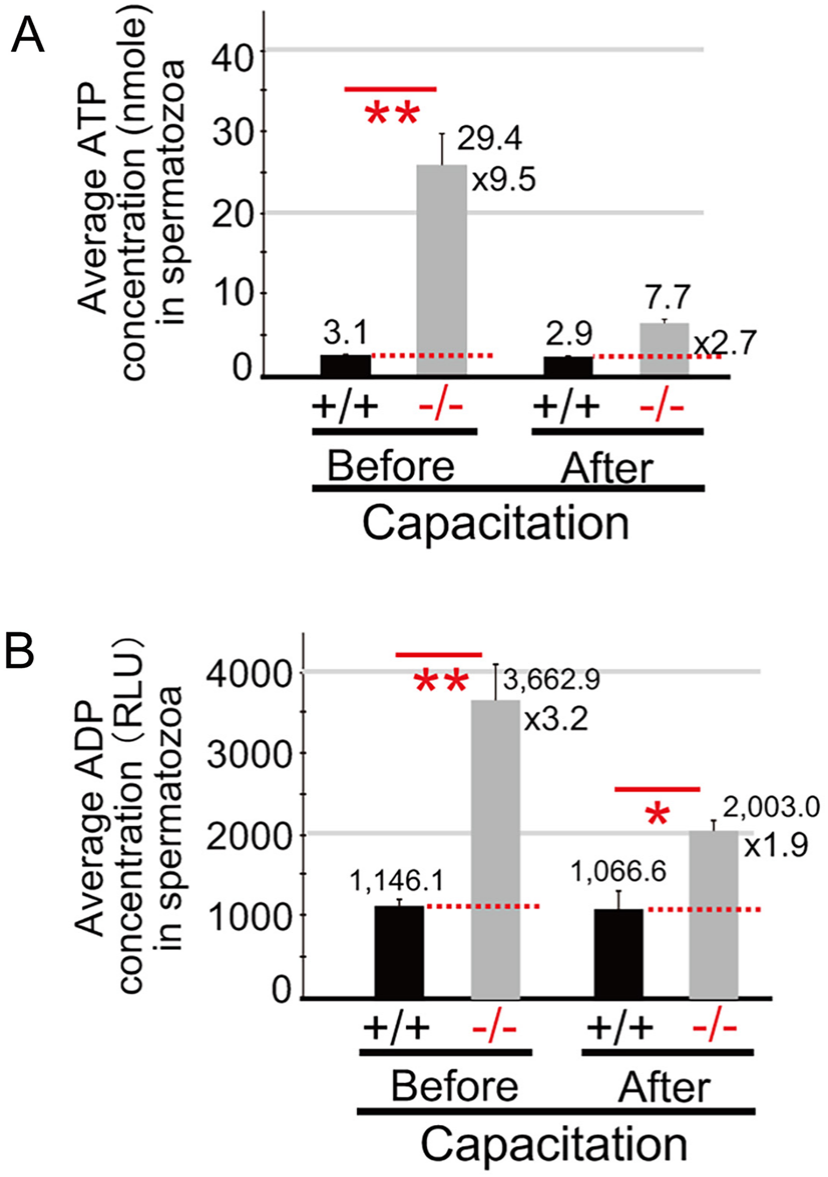
Average ATP and ADP concentrations in spermatozoa before and after capacitation. + / + : *Odf4*^+/+^. −/−: *Odf4*^−/−^. Sperm activation (capacitation) was performed in TYH medium. (**A**) Average ATP concentration (nmol per 1 × 10^4^ sperm). (**B**) Average ADP concentration (RLU per 1 × 10^4^ sperm). In *Odf4*^+/+^, both ATP and ADP concentrations before and after capacitation were almost constant: 3.1 and 2.9 nmol and 1,146.1 and 1,066.6 RLU, respectively. In contrast, in *Odf4*^−/−^, these values are not constant and show higher values than those of the control: 29.4 and 7.7 nmol and 3,662.9 and 2,003.0 RLU, respectively. Welch’s t test (two-tailed analysis). The raw data are shown in Fig. S9. *P < 0.05. **P < 0.01.

Considering the results obtained here, we next examined how ATP and ADP were produced in *Odf4*^−/−^ spermatozoa and how *Odf4*^−/−^ sperm energy was controlled. For these purposes, ATP and ADP concentrations between *Odf4*^+/+^ (control) and *Odf4*^−/−^ spermatozoa were compared (see Methods). When evaluating the results, it should be noted that sperm flagellar movement becomes vigorous after capacitation, as characterized by whiplash-like beating called hyperactivation^1^. In *Odf4*^+/+^ spermatozoa, both ATP and ADP were produced and consumed, where each ATP and ADP concentration before and after capacitation was almost even, showing values of 3.1 versus 2.9 nmol and 1,146.1 versus 1,066.6 RLU (arbitrary unit), respectively (Fig. 5 and Fig. S9). In contrast*,* in *Odf4*^−/−^ spermatozoa, both ATP and ADP were produced, but the ATP and ADP concentrations were both high and uneven before capacitation and low and uneven after capacitation, showing values of 29.4 versus 7.7 nmol and 3,662.9 versus 2,003.0 RLU, respectively (Fig. 5 and Fig. S9).

### Flagellar motility analysis

**Figure 6:**
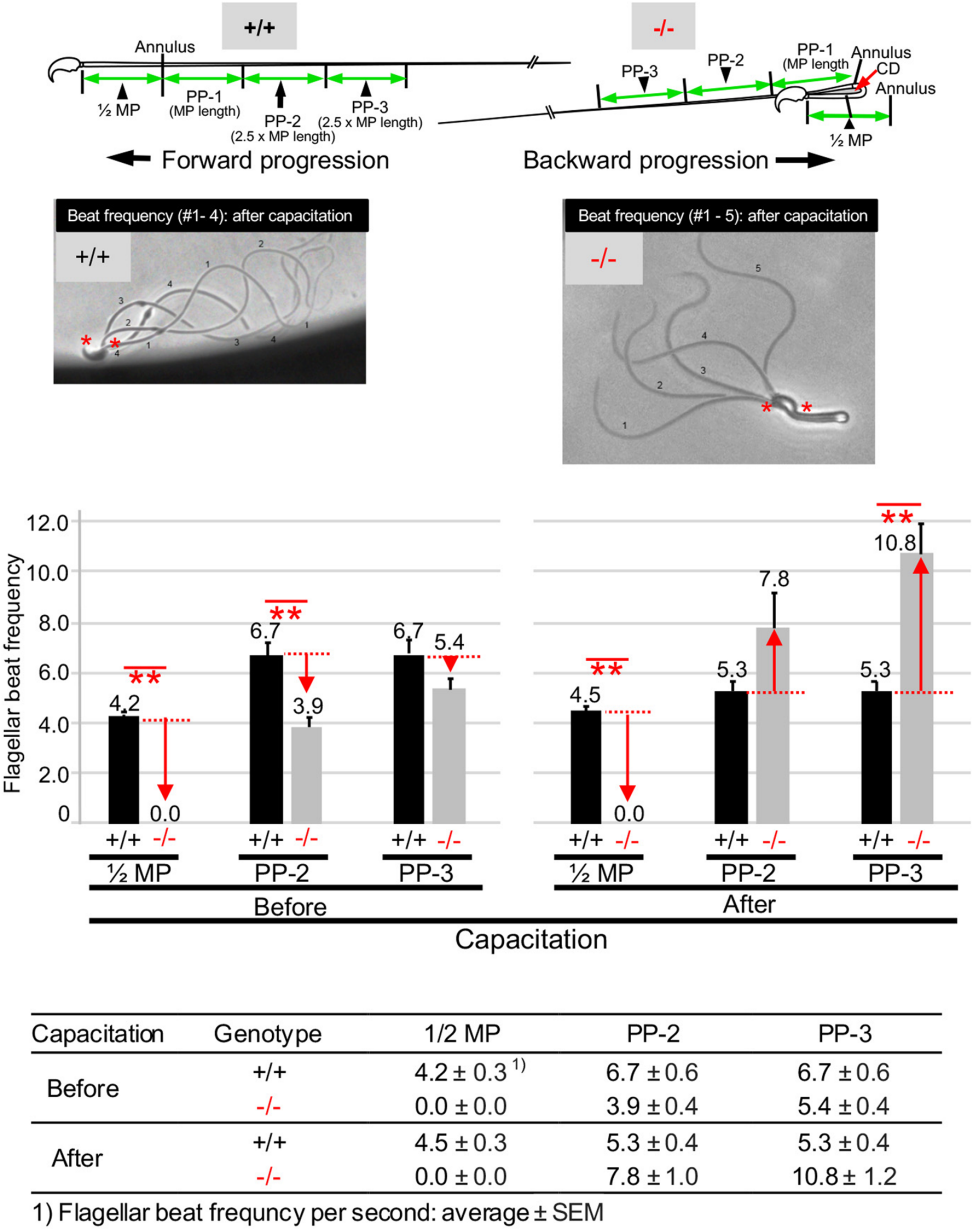
Flagellar beat frequency. + / + : *Odf4*^+/+^. −/−: *Odf4*^−/−^. Sperm activation (capacitation) was performed in TYH medium. The data are obtained from video and images analyzed using Premier Pro software (see Methods). (Top) Schematic images showing 3 positions of the flagellum for calculation: 1/2 MP (midpiece), PP (principal piece)-2 and PP-3 (arrowheads), where MP length is the distance from the neck to the annulus (approximately 22 μm). 1/2 MP = 1/2 × MP length. Below the schemes, representative still images analyzed are shown; the corresponding videos are shown in Videos S3 and S4. Midpiece movement is active with a symmetrical pattern in *Odf4*^+/+^ but immotile in *Odf4*^−/−^. Principal piece movement is active in both *Odf4*^+/+^ and *Odf4*^−/−^. The anteriormost and posteriormost regions of the head (*) were fixed at the same position during each analysis. (Middle) Graphs for flagellar beat frequency per second. *Odf4*^+/+^ flagellar movement is almost constant and regular: 4.2 and 4.5 at 1/2 MP and 6.7 and 5.3 at both PP-2 and PP-3 before capacitation and after capacitation, respectively. In contrast, in *Odf4*^−/−^, the midpiece is essentially immotile with null values (0.0), but the distal principal piece actively moves before capacitation and after capacitation with high values; the beat frequency is more active after capacitation (7.8 in PP-2 and 10.8 in PP-3) than before capacitation (3.9 in PP-2 and 5.4 in PP-3). Mann‒Whitney U test (two-tailed analysis) for + / + vs. −/− 1/2 MP before capacitation and after capacitation. Student’s t test (two-tailed analysis) for + / + vs. −/− PP-2 before capacitation and + / + vs. −/− PP-3 after capacitation. (Bottom) The table shows raw data. *N*males (*N*sperm): 4 (11) and 4 (14) for *Odf4*^+/+^ and *Odf4*^−/−^ before capacitation and 6 (17) and 4 (8) for *Odf4*^+/+^ and *Odf4*^−/−^ after capacitation, respectively. **P < 0.01.

Considering the result of Fig. 2D, we analyzed the details of *Odf4*^−/−^ flagellar motility by comparing the beat frequency at 3 points of the flagella (1/2, PP-2 and PP-3) in *Odf4*^−/−^ to *Odf4*^+/+^ spermatozoa (see Methods). The results are summarized in a table shown in Fig. 6 and described here in detail. The beat frequencies of the *Odf4*^+/+^ midpiece at 1/2 MP and the principal piece at PP-2 and PP-3 were consistently high both before and after capacitation, with values of 4.2 and 4.5 at 1/2 MP, 6.7 and 5.3 at PP-2, and 6.7 and 5.3 at PP-3, respectively. In contrast, the beat frequency of the *Odf4*^−/−^ midpiece at 1/2 MP was basically null both before and after capacitation, with values of 0 and 0, respectively. The beat frequency of the *Odf4*^−/−^ principal piece at PP-2 and PP-3 before capacitation was rather high, with values of 3.9 and 5.4, respectively, but these values were still lower than those of the *Odf4*^+/+^ midpiece. In contrast, the beat frequency of the *Odf4*^−/−^ principal piece at PP-2 and PP-3 after capacitation was quite high, where the values were 7.8 and 10.8, respectively; these values were higher than those of the *Odf4*^+/+^ principal piece. The raw data are shown in a table in Fig. 6 and in corresponding video clips (Videos S3 and S4 for *Odf4*^+/+^ and *Odf4*^−/−^, respectively).

### Demembranation assay

We also examined whether *Odf4*^−/−^ flagellar axial dynein ATPase functioned normally. Before describing the results, it should be noted that the Triton X-100 treatment disrupts the plasma and mitochondrial membranes but leaves the axoneme and cytoskeletal support intact^1^ and that in association with detergent treatment, cytosolic AK1 and AK2 disappear or are extracted, as shown by western blotting (Fig. 4). In addition, concomitantly with this assay, we examined how the presence or absence (disappearance) of flagellar CD affects the movement pattern, since CD disappears in response to Triton X-100 treatment. The experimental design and results are shown in a diagram (Fig. S10), also see Methods). Before demembranation, most of the control *Odf4*^+/+^ spermatozoa had straight flagella, no CD and moved forward (Video S5)*,* while the *Odf4*^−/−^ spermatozoa had bent flagella with a large CD and moved backward (Video S6). Next, we treated these spermatozoa with 0.1% Triton X-100, a treatment known as demembranation. As a result, soon after treatment, the demembranated spermatozoa of both *Odf4*^+/+^ and *Odf4*^−/−^ mice stopped moving and became immotile (Videos S7 and S8, respectively). Thereafter, we placed these demembranated spermatozoa in reactivating TYH medium (a modified Krebs–Ringer bicarbonate solution)^29^ that contains ATP to reactivate the spermatozoa; this is called reactivation. Both *Odf4*^+/+^ and *Odf4*^−/−^ spermatozoa immediately resumed active forward progression (Videos S9 and S10, respectively). During this reactivation assay, CDs were not observed in the Triton-treated *Odf4*^−/−^ flagella, and the CD-lost flagellar shape became rather straight (Video S10). These results are summarized in a table shown in Fig. S10.

### Environmental osmolarity changes do not affect *Odf4*^−/−^ flagellar angulation

Because it was previously reported that sperm flagellar angulation decreased as the in vitro environment osmolarity increased^22^, we examined how the osmolarity change in the environment of TYH medium affects *Odf4*^−/−^ flagellar angulation in an in vitro assay. For this purpose, we investigated flagellar status or angulation with increasing osmolarity—from 150 mOsm/kg, which corresponds to half the osmolarity of plasma, to 440 mOsm/kg, which is higher than uterine osmolarity (310 mOsm/kg). In *Odf4*^+/+^ spermatozoa, the percentage (%) of flagellar angulation with hairpin flagella decreased from approximately 60% to 20% as the osmolarity increased from 150 to 440 mOsm/kg, whereas in *Odf4*^−/−^ spermatozoa, the percentage (%) of flagellar angulation with hairpin flagella was always high (approximately 70%) at all osmolarity conditions, with approximately 90% bent tail spermatozoa (Figs S11A *Left* and S11B *Top*). Next, since it has been reported that sperm flagellar angulation decreases as osmolarity increases during sperm transit in the epididymis from approximately 330 mOsm/kg in the testis and the initial segment of the caput^23^ to approximately 415 mOsm/kg in the cauda epididymis^22^, we examined whether *Odf4*^−/−^ flagellar angulation changes when sperm passes from the caput to the cauda epididymis in vivo. As shown in Figs S11A *Right* and S11B *Bottom*, in the control *Odf4*^+/+^ spermatozoa, the percentage (%) of bent tail (V shape and hairpin) flagellar angulation was high in the caput and corpus epididymis at nearly 80%, and then it decreased to nearly 30% as they passed toward the distal epididymis. In contrast, in *Odf4*^−/−^ spermatozoa, the percentage (%) of bent tails was consistently high (70–95%) in any region of the epididymis, where the percentage (%) of hairpin tails increased from approximately 30% to approximately 80% in the epididymis.

### Rescue experiments

**Figure 7:**
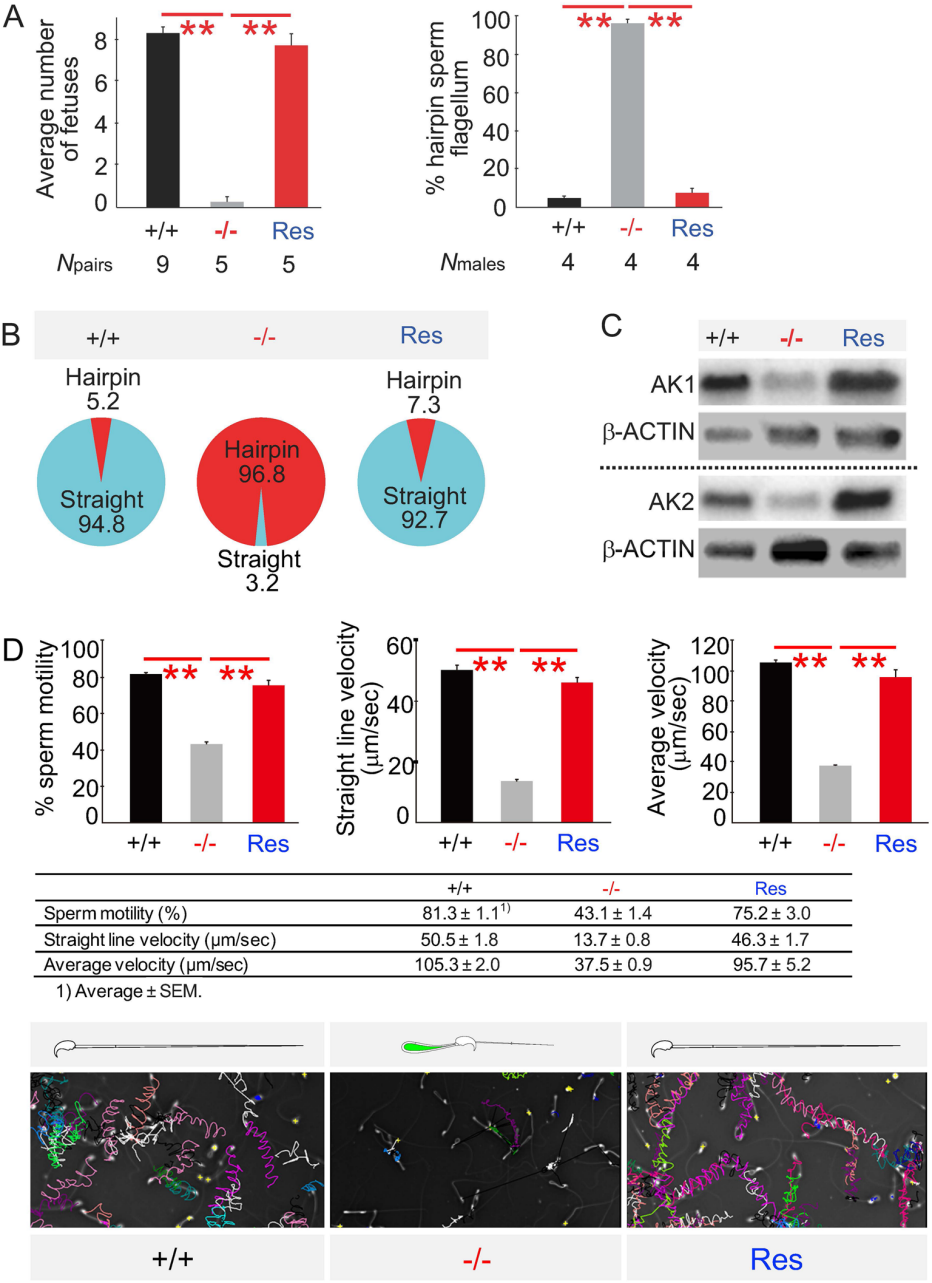
Rescue experiments. + / + :*Odf4*^+/+^. −/−:*Odf4*^−/−^. Res: Rescue. The rescue male was made by insertion of the *Odf4-Egfp* gene into *Odf4*^−/−^ (Fig. S12) to show that rescued Tg(Odf4- Egfp)*Odf4*^−/−^ males restore a healthy reproductive phenotype to *Odf4*^−/−^ males. (**A**) (Left) Fertility rate: 8.3 ± 0.3 (+ / +), 0.3 ± 0.3 (−/−), and 7.8 ± 0.6 (Res). (Right) Percentage (%) postcopulatory hairpin sperm flagella: 5.0 ± 0.8 (+ / +), 96.0 ± 2.4 (−/−) and 7.5 ± 2.7 (Res). Steel–Dwass test (nonparametric test). (**B**) Restoration of percentage (%) of hairpin flagella in cauda epididymal spermatozoa. (**C**) Cropped images of western blotting with antibodies against AK1 (Top) and AK2 (Bottom). Both AK1 and AK2 signals were detected in rescued spermatozoa as strong as in *Odf4*^+/+^ spermatozoa. Further cropped images are shown in Fig S12D. These original full-length images with different exposure times are shown in the supplementary SD16. (**D**) (Top) Sperm movement analysis by SMAS. (Left) Percent (%) sperm motility. (Middle) Straight line velocity. (Right) Average velocity. These movements were significantly lower in *Odf4*^−/−^ mice than in *Odf4*^+/+^ mice but were restored in rescued males. Welch’s t test (two-tailed analysis). (Middle) Table shows raw data. The motility level of the rescued spermatozoa is almost the same as that of the wild-type (+ / +) spermatozoa. (Bottom) Representative tracts (colors for individual sperm). *N*males (*N*sperm): 5 (10,406), 5 (9,030) and 3 (12,517) for *Odf4*^+/+^, *Odf4*^+/-^ and rescue males, respectively. **P < 0.01.

To confirm the importance of *Odf4* and ODF4 in vivo, we tested whether rescue mouse Tg(Odf4-Egfp)*Odf4*^−/−^ spermatozoa can rescue the phenotype caused by the *Odf4* gene deletion (see Methods). The evidence of Tg generation was shown by genomic PCR and western blotting (Fig. S12). As a result, the rescue Tg males exhibited complete restoration of all parameters and events examined: infertility rate, percentage (%) hairpin sperm flagellum in the cauda epididymal and postcopulatory spermatozoa, reduction of AK1 and AK2, and reduction of sperm motility (Fig. 7, Fig. S12. The restored values obtained in these assays were almost equal to the levels obtained for the control *Odf4*^+/+^ spermatozoa.

## Discussion

**Figure 8:**
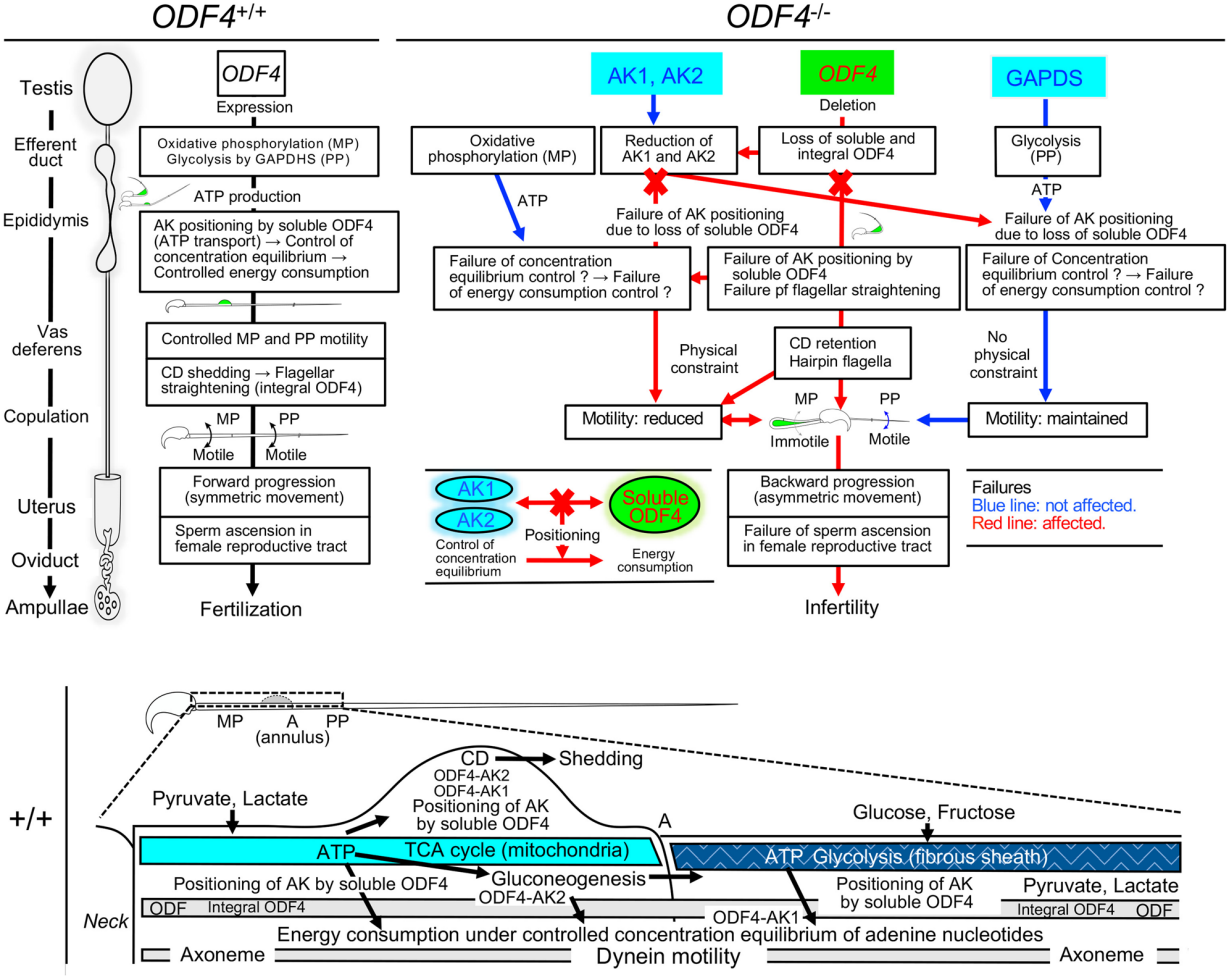
Summary of the present data (Top) and possible interplay of ODF4, AK1 and AK2 in *Odf4*^+/+^ spermatozoa (Bottom). (Top, left) In *Odf4*^+/+^ spermatozoa, soluble ODF4 associates with AK1 and AK2. Flagellar shape (straightening) and forward progression are controlled. (Top, Right) In *Odf4*^−/−^ spermatozoa, *Odf4* deletion primarily causes the loss of integral and soluble ODF4 in the testis and secondarily causes reduction of AK1 and AK2 in mature sperm flagella; eventually, a hairpin flagellum with a large cytoplasmic droplet (CD) appears and shows backward progression, leading to male infertility. A detailed explanation is provided in the Discussion. (Bottom) The energy production and consumption system normally works under controlled conditions of concentration equilibrium of adenine nucleotides, where ODF4, AK1 and AK2 are present. The ATP produced by the TCA cycle is normally transported, mainly by the ODF4-AK2 complex, to the energy-consuming site (CD) and axonemal dynein in the midpiece, while the ATP produced by glycolysis is mainly transported by the ODF4-AK1 complex to the energy-consuming site (axonemal dynein in the principal piece). The transported ATP will help shed the CD and maintain movement in the midpiece and principal piece. *MP* midpiece, *PP* principal piece, Top, Bottom: drawn by Toshimori, K.

*Odf4* deletion primarily causes the loss of ODF4 in *Odf4*^−/−^ testes and secondarily reduces the amount of AK1 and AK2 in *Odf4*^−/−^ sperm flagella; eventually, *Odf4*^−/−^ males are infertile. Herein, we present our explanations and possible interplays of soluble ODF4, AK1 and AK2 in *Odf4*^+/+^ spermatozoa based on the summary of our studies (Fig. 8). First, the result of the double Tg(Odf2-mCherry,Odf4-Egfp) expression experiment showing that ODF4 is more broadly distributed in the flagella than the cytoskeletal protein ODF2 (Fig. 1E) morphologically implies that ODF4 has some role(s) in addition to that of integral ODF4. This implication is supported by the western blotting results showing that there is a soluble form of ODF4 (soluble ODF4). Soluble ODF4 can be readily extracted by RIPA buffer containing 1% Nonidet 40; Nonidet 40 is a milder detergent than 6 M urea, which is conventionally used to extract flagellar cytoskeletal proteins, such as ODF1 and ODF2. Integral ODF4 is traditionally known as an integral structural component that associates with other ODF components^1^ (NCBI). Another western blotting result showing that AK1 and AK2 in *Odf4*^−/−^ mice are detected in testicular samples but are significantly reduced in mature sperm extracts (Fig. 4A) indicates that AK1 and AK2 are produced in *Odf4*^−/−^ testes, but both AK1 and AK2 are not well mobilized in mature *Odf4*^−/−^ spermatozoa owing to the loss of ODF4. The results of *Odf4*^−/−^ sperm motility reduction (Fig. 2D) and coimmunoprecipitation assays of *Odf4*^+/+^ sperm samples (Fig. 4B) suggest that sperm flagellar motility is achieved through ODF4 interaction with AK1 and AK2. Considering all these implications together with the results that ODF4, AK1 and AK2 are distributed to *Odf4*^+/+^ whole flagella, including the CD, but AK1 and AK2 are hardly detected in *Odf4*^−/−^ spermatozoa (Figs. 4A and S3), soluble ODF4 can bind to AK1 and AK2 and function in the cytosol outside the ODFs. Thus, soluble ODF4 is involved in the correct positioning of AK1 and AK2 in the flagellum. It is also presumable that the ODF4-AK1-AK2 complexes shuttle ATP to the flagellar cytoplasm to control the concentration equilibrium of adenine nucleotides for CD elimination. Thus, the interaction of ODF4, AK1 and AK2 in spermatozoa will be a prerequisite for energy metabolism for sperm flagellar shape (straightening) and movement.

Regarding sperm flagellar energy metabolism, ATP and ADP assays (Fig. 5 and Fig. S9) also indicated that in *Odf4*^−/−^ spermatozoa, ATP is produced but not correctly transported to the consuming sites owing to the absence of ODF4 and the reduction of AK1 and AK2; thus, the consumption of ATP is not well controlled. In fact, the concentrations of ATP and ADP in *Odf4*^−/−^ spermatozoa were random, with high and uneven averages throughout capacitation. In contrast, in *Odf4*^+/+^ spermatozoa, the produced ATP and ADP are normally positioned, transported, consumed and balanced under controlled conditions, owing to the normal association of ODF4 with AK1 and AK2. In fact, the concentrations of ATP and ADP were always low, with almost even averages throughout capacitation.

Regarding the *Odf4*^−/−^ flagellar movement as shown in Figs. 2, 5, 6, it is thought that the energy (ATP) produced by oxidative phosphorylation in the midpiece, where huge CD is present (not eliminated), is not enough to shed the CD and maintain powerful movement. In addition, the remaining large CD mechanically disturbs the movement and straightening of the midpiece, where the reduced integral ODF4 may also disturb the straightening of the midpiece. In contrast, the energy (ATP) produced by glycolysis in the principal piece is still enough to maintain movement, whereas the absence of CD does not disrupt movement. However, the precise mechanism of how AK1 and AK2 are involved in CD shedding and flagellar movement is currently unclear. In addition, until now, there has been no evidence about structures and molecules for CD shedding throughout spermatogenesis to sperm maturation in the epididymis^1^. Although AK8 is reported to be present in sperm flagella^17^, whether AK8 is present in *Odf4*^−/−^ flagella is unclear because a specific antibody against AK8 is not currently available.

The reduction in *Odf4*^−/−^ midpiece motility was not caused by the disorder of axial dynein because *Odf4*^−/−^ dynein was found to be normal (Fig. 3D) and because freely available ATP in the reactivation medium was normally consumed, as shown by the demembranation assay (Fig. S10, Video S10). In addition, the demembranation assay also showed that most CD-lacking *Odf4*^−/−^ flagella became rather straight shaped and moved forward after CD loss, indicating that CD shedding is essential for flagellar straightening and forward progression. *Odf4*^−/−^ flagellar bending is induced neither by environmental osmolarity changes, as shown in Fig. S11, nor by deficiencies in other genes, such as aquaporin genes (*AQP3*, *AQP7,* and *AQP8*)^22^, solute carrier genes (*SlC26A8*, *SlC22A14*)^24,25^ and annulus-related genes (*SEPTIN4* and *SEPTIN 7*)^25^, because all of these gene products were present in *Odf4*^−/−^ spermatozoa (Fig. 4).

Last, since all abnormalities and male infertility caused by *Odf4* deletion described above were reversed by *Odf4* restoration (Fig. 8), the rescue experiment confirmed the importance of ODF4, AK1, and AK2 in vivo. Thus, our study reports the contribution of the newly identified ‘soluble ODF4’ associated with AK1 and AK2 to fertility and provides its relationship with sperm flagellar shape (straightening) and movement. This report will open a new window to understanding the molecular mechanism of fertility and infertility. Our findings also bestow knowledge in the field of human diseases, since it is becoming clear that many of the etiologies of male infertility are due to inherited genetic variations^26^.

## Materials and methods

### Ethical approval and animals

All procedures were performed in accordance with the institutional guidelines for animal research and approved by the Animal Care and Use Committee of Chiba University (#A30-080) for C57BL/6JJmsSlc (B6), B6D2F1 (BDF1) and ICR mice (Japan SLC). This study was carried out in compliance with the ARRIVE guidelines.

### Antibodies

The antibodies and chemicals used are described in Table S1.

### Generation of KO mice, B6(Cg)-*Odf4*^em1^: *Odf4*-null (*Odf4*^−/−^) mice

The CRISPR target sequence of the mouse *Odf4* gene was determined in exon 1 using the online sgRNA design tool CRISPRdirect software (https://www.crisper.dbcls.jp): TGAATGAGGAAGAATCCGAGAGG (protospacer adjacent motif sequences are underlined). The sgRNA was synthesized using synthetic oligonucleotides (5’-TAATACGACTCACTATAGTGAATGAGGAAGAATC-3’ and 5’-TTCTAGCTCTAAAACCTCGGATTCTTCCTCATT-3’). The CRISPR/Cas9 mixture containing 50 ng/μl sgRNA and 100 ng/μl Cas9 nuclease (Thermo Fisher Scientific) was injected into BDF1 pronuclear stage embryos, which were cultured into two-cell embryos and transferred into the oviducts of ICR pseudopregnant female mice; 26 pups (F0) were born. Genomic DNA was used for PCR amplification with gene-specific primers for the detection of mutations (Table S2). The sequences of the clones were determined by sequencing. Three males and one female among the F0 mice exhibited frameshift mutations on both alleles. The males were infertile, but the female was fertile and produced pups (F1) after crossing with a wild-type partner. *Odf4*-homozygous (*Odf4*-null or ^−/−^) mice were produced by crossing heterozygous (+ /-) mice.

### Generation of Tg mice

All Tg mice were produced according to a previously reported method^27^. **Double-Tg**(**Odf2-mCherry,Odf4-Egfp**)** mice.** First, we generated Tg(Odf4-Egfp)*Odf4*^+*/*+^ mice and Tg(Odf2-mCherry)*Odf4*^+*/*+^ mice separately (Fig. 1C). The mouse *Odf4* cDNA was fused with a linearized pCM*-Egfp* vector containing the mouse Calmegin promoter^28^, which was made from the pCM*-Eqtn-Egfp* vector^27^. The mouse *Odf2* cDNA was cloned into the pmCherry vector (Clontech), and then the *Odf2-mCherry* fragment was cloned into the pCM vector (pCM-*Odf2-mCherry*). The transgenes excised from pCM-*Odf4-Egfp* and pCM-*Odf2-mCherry* were injected into fertilized eggs of C57BL/6 mice. Incorporation of the transgene was detected via PCR analysis using primer set A for Tg(Odf4-Egfp) and primer set B for Tg(Odf2-mCherry), as shown in Table S2. Tg(Odf4-Egfp) mice were crossed with Tg(Odf2-mCherry) mice to generate double Tg(Odf2-mCherry,Odf4-Egfp) mice (Fig. 1C). Tg(Eqtn-Egfp)*Odf4*^+*/*+^ mice were generated as previously reported^27^. Tg(Eqtn-Egfp)*Odf4*^−/−^ was generated by crossing Tg(Eqtn-Egfp)*Odf4*^+*/*+^ males with *Odf4*^−/−^ females. **Rescue Tg males.** Tg(Odf4-Egfp)*Odf4*^+*/*+^ males were mated with *Odf4*^−/−^ females to obtain heterozygous Tg(Odf4-Egfp)*Odf4*^+*/-*^ mice. Tg(Odf4-Egfp)*Odf4*^+*/-*^ males and females were mated to obtain Tg(Odf4-Egfp)*Odf4*^−/−^ mice for the rescue experiment.

### RT‒PCR

Total RNA was isolated from various tissues (cerebrum, heart, lung, liver, spleen, kidney, small intestine, testis, epididymis) of adult C57BL/6 mice and testes of 1-, 2-, 3-, 4- and 5-week-old ICR mice. PCR was performed using Odf4-specific primer pairs (Table S2) at 94 °C for 2 min and 30x (94 °C for 20 s, 55 °C for 30 s, and 72 °C for 60 s). Gapdh (D-glyceraldehyde-3-phosphate dehydrogenase)-specific primers (Table S2) were used as controls.

### SDS‒PAGE and western blotting

These methods were performed as previously reported^29^, with slight modifications. For ODF1, ODF2, TEKTIN4 and β-TUBULIN, spermatozoa or testes were minced and centrifuged, and the supernatants were incubated twice in a buffer including 2% Triton X-100 and 5 mM DTT containing protease inhibitor cocktail for 15 min on ice and then lysed in lysis buffer containing 6 M urea and 5 mM DTT for 20 h on ice. For ODF4, AK1 and 2, CATSPER3, SEPTIN4 and 7, GAPDH, GAPDS, AQP3,7 and 8, SLC22A14 and β-ACTIN, β-TUBULIN, samples were recovered from the epididymis by pricking out with needles or from the testicular seminiferous tubules and then directly put into RIPA buffer (50 mmol/L Tris–HCl buffer (pH 7.6), 150 mmol/L NaCl, 1% Nonidet P40 substitute, 0.5% sodium deoxycholate, 0.1% SDS) without washing to solubilize for 2 h on ice. These lysates were routinely used for experiments performed in at least triplicate (*N*sample = 3).

### IP

The experimental pair included *Odf4*^+/+^ Tg(Odf4-Egfp) and *Odf4*^−/−^ (control) spermatozoa**.** These samples were similarly extracted in RIPA buffer, as described above for western blotting. The same amount of samples for each pair was subjected to IP, which was performed using a Dynabeads™ Protein G Immunoprecipitation Kit with a mouse monoclonal antibody against GFP (B2: IgG_2a_, Santa Cruz Biotechnology Inc.). The obtained samples were subjected to SDS‒PAGE and western blotting.

### Fertility test

Male mice were housed with females for at least 2 months to determine whether litters were obtained. The number of pups produced was checked every morning.

### Morphology and motility assays

Spermatozoa were prepared by squeezing them out from the caput, corpus and cauda epididymis of *Odf4*^+/+^ (+ / +) and various *Odf4*^−/−^ (−/−) and Tg males. They were processed as previously reported^21^. Some spermatozoa were briefly treated with 1% saponin for immunofluorescence to visualize the internal components.

### Postcopulatory spermatozoa

These spermatozoa were collected from the B6D2F1 female uterus after copulation with the males and analyzed by light microscopy.

### Light microscopy, immunofluorescence, and DIC

Light microscopic analyses were performed as previously reported^21^.

### TEM and immunoelectron microscopy

TEM^21^ and immunoelectron microscopy^30^ were performed as previously reported, with some modifications as follows. Spermatozoa removed from Tg(Odf4-Egfp) cauda epididymis were treated with 1% saponin in TYH followed by an antibody against GFP conjugated with 5 nm gold.

### SMAS

Fresh spermatozoa were collected from the cauda epididymis. After incubation in a CO_2_ incubator for 30 min, sperm motility and other characteristic parameters were quantitatively analyzed using an automated SMAS (DITECT Co. Ltd.), as previously described^29^.

### Comparative analysis of sperm ascension in the female reproductive tract

Tg(Eqtn-Egfp)Odf4^+/+^ (control) and Tg(Eqtn-Egfp)Odf4^−/−^ males were used as experimental pairs. These Tg spermatozoa were traced and visualized by taking photographs under a Leica M165 FC fluorescence stereomicroscope, according to previously reported methods^29^.

### ATP and ADP concentration measurements

Measurements were performed for each ATP concentration (nmol/1 × 10^4^ sperm) and ADP concentration (RLU/1 × 10^4^ sperm) before and after capacitation in *Odf4*^+*/*+^ (control) and *Odf4*^−/−^ spermatozoa, where RLU is an arbitrary unit (read luminescence on the luminometer) read by a luminometer with a Filter Max F5 (Molecular Device). The ATP concentration was measured using an Intracellular ATP assay kit ver. 2 (IC2-100 assay, Toyo B-Net.) with some modifications for nmol/1 × 10^4^ sperm, referring to a previously reported method^10^. The ADP concentration (RLU) was measured using an EnzyLight™ ADP/ATP Ratio Assay Kit (ELDT-100; BioAssay Systems).

### Demembranation assay

This assay was performed according to previously reported methods^31^, with some modifications as described below and shown in Fig. S10. First, spermatozoa were allowed to swim in IVF dishes containing TYH medium in a CO2 incubator for 90 min. Then, for demembranation assays, 5 μl of the sperm suspension was transferred into 45 μl of the extraction solution containing 0.1% (v/v) Triton X-100, 200 mM sucrose, 25 mM glutamic acid, (25 mM KOH), 1 mM DTT, and 20 mM HEPES–NaOH (pH 7.4) in a test tube, transferred to 35 mm dishes and covered with mineral oil for video recordings. Then, approximately 120 min after the start of the initial incubation, a reactivation assay was performed as follows. Five microliters of the mixture containing the demembranated spermatozoa was transferred into 45 μl of the reactivation solution containing 200 mM sucrose, 25 mM glutamic acid, (25 mM KOH), 1 mM DTT, 1 mM EGTA, 4 mM MgSO4, 3 mM ATP, and 20 mM HEPES–NaOH (pH 7.4) in a test tube at 37 °C. Then, 5 μl of the incubated sperm suspension was similarly placed on a stage of DMi8 stereoscopy for video recording.

### Video creation using a high-speed camera

Flagellar movement was recorded for analyses at 30 fps for 15 s under a DMi8 stereomicroscope equipped with neo PLAN × 20 or × 40 objective lenses and an MC170 HD camera (Leica). The original high-speed video (avi) was recorded at 1,027 × 1,024, Fps 1,000–1,200, SS 1/1,000 using DIPP-Motion V (ver. 1.1.35) software (DITECT) under a DMi8 stereomicroscope equipped with a HAS-U2 camera (DITECT). The videos were used to analyze movement using Premier Pro software (Adobe) and Adobe Photoshop CC 2019 (Adobe), depending on the purpose. In addition, images were taken from the video. The length of the subject was calculated in pixel mode, e.g., for a head length of approximately 7 μm. The flagellar beat frequency (F) is expressed by the following formula: F = 1 T, where F is the beat frequency (Hz) and T is the time (seconds) that were taken for videos; the raw data were recalculated as the beat frequency per second and are shown in Fig. 6.

### Environmental osmolarity change effect

The osmolarity of the TYH medium for the sperm samples was adjusted to 150, 310, 380, and 440 mOsm/kg using 1 M NaCl by calculation with a digital micro osmometer (Genotec Osmomat 3000); this condition was used for the in vitro assay. Spermatozoa passing through the epididymis were recovered from each region from the caput to the cauda epididymis (Fig. S11) and immediately subjected to experiments without any treatment. Analyses (quantification) were performed after obtaining images according to the criteria of flagellar shapes shown at the top of the panel in Fig. S11.

### Statistics

Data are shown as the mean ± standard error of the mean (SEM) after statistical analysis (*P < 0.05 or **P < 0.01) by the method written in each graph (Figs. 2, 3, 5, 6, 7).

## Supplementary Information

Supplementary Information 1.

Supplementary Information 2.

Supplementary Information 3.

Supplementary Video 1.

Supplementary Video 2.

Supplementary Video 3.

Supplementary Video 4.

Supplementary Video 5.

Supplementary Video 6.

Supplementary Video 7.

Supplementary Video 8.

Supplementary Video 9.

Supplementary Video 10.

## Data Availability

The original contributions presented in this study are publicly available. KO and Tg mice used in this study have been deposited into the Mouse Genome Informatics (MGI) [J: 327,405 (PubMed ID), MGI 7,331,638 for *Odf4*-null (*Odf4*^−/−^), and MGI 7,331,643 for Tg(Odf4-Egfp) and Tg(Odf2-mCherry)]. The datasets that support the findings of this study are available here: “figshare” at https://figshare.com. All other data are available in the main text and supplementary information.

## References

[1] Toshimori, K. & Eddy, E. M. in *Knobil and Neill's Physiology of Reproduction* Vol. 1 (eds T.M. Plant & A.J. Zeleznik) Ch. 3, 99–148 (Academic Press, 2014).

[2] WHO. WHO laboratory manual for the Examination and processing of human semen. *Fifth EDITION* (2010). PMC4975259 27683423

[3] Nakamura Y (2002). Molecular cloning and characterization of oppo 1: A haploid germ cell-specific complementary DNA encoding sperm tail protein. Biol. Reprod..

[4] Kitamura K (2003). Molecular cloning and characterization of the human orthologue of the oppo 1 gene encoding a sperm tail protein. Mol. Hum. Reprod..

[5] Cooper TG (2011). The epididymis, cytoplasmic droplets and male fertility. Asian J. Androl..

[6] Hermo L, Oliveira RL, Smith CE, Au CE, Bergeron JJM (2019). Dark side of the epididymis: Tails of sperm maturation. Andrology.

[7] Yuan S, Zheng H, Zheng Z, Yan W (2013). Proteomic analyses reveal a role of cytoplasmic droplets as an energy source during epididymal sperm maturation. PLoS ONE.

[8] Dzeja P, Terzic A (2009). Adenylate kinase and AMP signaling networks: metabolic monitoring, signal communication and body energy sensing. Int. J. Mol. Sci..

[9] Leigh MW (2009). Clinical and genetic aspects of primary ciliary dyskinesia/Kartagener syndrome. Genet. Med..

[10] Miki K (2004). Glyceraldehyde 3-phosphate dehydrogenase-S, a sperm-specific glycolytic enzyme, is required for sperm motility and male fertility. Proc. Natl. Acad. Sci. U S A.

[11] Mukai C, Okuno M (2004). Glycolysis plays a major role for adenosine triphosphate supplementation in mouse sperm flagellar movement. Biol. Reprod..

[12] Krisfalusi M, Miki K, Magyar PL, O'Brien DA (2006). Multiple glycolytic enzymes are tightly bound to the fibrous sheath of mouse spermatozoa. Biol. Reprod..

[13] Atkinson DE (1968). The energy charge of the adenylate pool as a regulatory parameter. Interaction with feedback modifiers. Biochemistry (Mosc).

[14] Goldberg RN, Tewari YB, Bhat TN (2004). Thermodynamics of enzyme-catalyzed reactions–a database for quantitative biochemistry. Bioinformatics.

[15] Cao W, Haig-Ladewig L, Gerton GL, Moss SB (2006). Adenylate kinases 1 and 2 are part of the accessory structures in the mouse sperm flagellum. Biol. Reprod..

[16] Xie M (2020). Adenylate kinase 1 deficiency disrupts mouse sperm motility under conditions of energy stress. Biol. Reprod..

[17] Vadnais ML (2014). Adenine nucleotide metabolism and a role for AMP in modulating flagellar waveforms in mouse sperm. Biol. Reprod..

[18] Pucar D (2000). Compromised energetics in the adenylate kinase AK1 gene knockout heart under metabolic stress. J. Biol. Chem..

[19] Dzeja PP, Bast P, Pucar D, Wieringa B, Terzic A (2007). Defective metabolic signaling in adenylate kinase AK1 gene knock-out hearts compromises post-ischemic coronary reflow. J. Biol. Chem..

[20] Janssen EM, van Oosterhout AJ, Nijkamp FP, van Eden W, Wauben MH (2000). The efficacy of immunotherapy in an experimental murine model of allergic asthma is related to the strength and site of T cell activation during immunotherapy. J. Immunol..

[21] Ito C (2019). Odf2 haploinsufficiency causes a new type of decapitated and decaudated spermatozoa, Odf2-DDS, in mice. Sci. Rep..

[22] Chen Q (2011). Aquaporin3 is a sperm water channel essential for postcopulatory sperm osmoadaptation and migration. Cell Res..

[23] Tuck RR, Setchell BP, Waites GM, Young JA (1970). The composition of fluid collected by micropuncture and catheterization from the seminiferous tubules and rete testis of rats. Pflugers Arch..

[24] Touré A (2007). The testis anion transporter 1 (Slc26a8) is required for sperm terminal differentiation and male fertility in the mouse. Hum. Mol. Genet..

[25] Maruyama SY (2016). A critical role of solute carrier 22a14 in sperm motility and male fertility in mice. Sci. Rep..

[26] Lores P (2018). Homozygous missense mutation L673P in adenylate kinase 7 (AK7) leads to primary male infertility and multiple morphological anomalies of the flagella but not to primary ciliary dyskinesia. Hum. Mol. Genet..

[27] Ito C (2013). Integration of the mouse sperm fertilization-related protein equatorin into the acrosome during spermatogenesis as revealed by super-resolution and immunoelectron microscopy. Cell Tissue Res..

[28] Watanabe D (1995). Characterization of the testis-specific gene 'calmegin' promoter sequence and its activity defined by transgenic mouse experiments. FEBS Lett..

[29] Ito C (2018). Deletion of Eqtn in mice reduces male fertility and sperm-egg adhesion. Reproduction.

[30] Ito C (2010). Tetraspanin family protein CD9 in the mouse sperm: unique localization, appearance, behavior and fate during fertilization. Cell Tissue Res..

[31] Gibbons BH, Gibbons IR (1973). The effect of partial extraction of dynein arms on the movement of reactivated sea-urchin sperm. J. Cell Sci..

