# Supplementary material for: The association of ODF4 with AK1 and AK2 in mice is essential for fertility through its contribution to flagellar shape
Source: Sci Rep. 2023 Feb 20;13:2969. doi: 10.1038/s41598-023-28177-z (PMC9941515; doi:10.1038/s41598-023-28177-z)
Supplement: Supplementary file 1 — Supplementary Information 1. [file 41598_2023_28177_MOESM1_ESM.pdf]

Supplementary information

The association of ODF4 with AK1 and AK2 in mice is essential for fertility through its contribution to flagellar shape

Chizuru Ito[1,#,*], Tsukasa Makino[2], Tohru Mutoh[1], Masahide Kikkawa[2], and Kiyotaka Toshimori[1,3,#,*]

[1]Department of Functional Anatomy, Reproductive Biology and Medicine, Graduate School of Medicine, Chiba University, Chiba, 260-8670, Japan. [2]Department of Cell Biology and Anatomy, Graduate School of Medicine, The University of Tokyo, Tokyo, Japan. [3]Future Medicine Research Center, Chiba University, Chiba, 260-8670, Japan

[#] C.I., and K.T. contributed equally to this work.

*Correspondences and requests for materials should be addressed to C.I. (email: chizuru@faculty.chiba-u.jp) or K. T. (email: ktoshi@faculty.chiba-u.jp)

Supplementary Tables

Table S1. Antibodies and chemicals used in this study.

Antibodies (characteristics) or chemicals: catalog number	Host[1)]	Dilution (Western blotting)	Dilution (IF, IEM and IP)[2)]	Reeference or source
Anti-AK1 (E-8): sc-365316	M	1/250 of the stock solution (0.2 mg/ml)	1/100 of the stock solution	Santa Cruz Biotechnology, Inc. (Dallas, TX)
Anti-AK2 (F-2): sc-374095	M	1/250 of the stock solution (0.2 mg/ml)	1/100 of the stock solution	Santa Cruz Biotechnology, Inc. (Dallas, TX)
Anti-AQUAPORIN3: ab125219	R	1/250 of the stock solution (0.5 mg/ml)		abcam (Tokyo, Japan)
Anti-AQUAPORIN 7 (D-12): sc-376407	M	1/100 of the stock solution (0.2 mg/ml)		Santa Cruz Biotechnology, Inc. (Dallas, TX)
Anti-AQUAPORIN 8: PA5-97109	R	1/250 of the stock solution (2.11 mg/ml)		Thermo Fisher Scientific (Waltham, MA)
Anti-β–ACTIN: M177-3	M	1/2000 of the stock solution (1 mg/ml)		Medical & Biological labolatories CO.,LTD. (Nagoya, Japan)
Anti-β-TUBULIN: 014-25041	M	1/10,000 of the stock solution (0.5 mg/ml)		FUJIFILM Wako Pure Chemicals (Osaka, Japan)
Anti-CATSPER3: ab197924	R	1/250 of the stock solution (1.4 mg/ml)		abcam (Tokyo, Japan)
Anti-GAPDH: 014-25524	M	1/2500 of the stock solution (0.5 mg/ml)		FUJIFILM Wako Pure Chemicals (Osaka, Japan)
Anti-GAPDS: ab153802	R	1/800 of the stock solution (0.91 mg/ml)		abcam (Tokyo, Japan)
Anti-GFP Ab (B 2) : sc-9996	M	1/250 of the stock solution (0.2 mg/ml)	1/100 of the stock solution (0.5 mg/ml) 0.2mg/ml for IP	Santa Cruz Biotechnology, Inc. (Dallas, TX)
Anti-ODF1 (E-11) : sc-390152	G	1/1,000 of the stock solution (0.2 mg/ml)		Santa Cruz Biotechnology, Inc. (Dallas, TX)
Anti-ODF2: 12058-1-AP	R	1/5,000 of the stock solution (0.53 mg/ml)		Proteintech Group (Rosemont, Ill)
Anti-ODF4: Custom-made	R		1/100 of the stock solution (1.4 mg/ml)	Custom-made
Anti-ODF4: bs-13615R	R	1/100 of the stock solutuin (1mg/ml)		Bios Inc (Boston, MA)
Anti-SEPTIN4: ab-166788	G	1/250 of the stock solution (0.5 mg/ml)		abcam (Tokyo, Japan)
Anti-Human SEPTIN7 (C): 18991	R	1/2000 of the stock solution (0.1 mg/ml)	1/100 of the stock solution	Immuno-BiologicalLaboratories Co, Ltd. (Tokyo,Japan)
Anti-SLC22A14: Custom-made (gift)	R	1/1000 of the stock solution (1 mg/ml; original gift)		Maruyama et al (2016)
Anti-TEKTIN4: Custom-made (gift)	R	1/250 of the stock solution of the original gift		Iida et al (2006)[2)]
Anti-rabbit IgG (Alexa Fluor 488): A-11008	G		1/2,000 of the stock solution (2 mg/ml)	Thermo Fisher Scientific (Waltham, MA)
Anti-goat IgG (Horseradish peroxidase): 705-035-003	D	1/20,000 of the stock solution (0.4 mg/ml)		Jackson Immunoresearch (West Grove, PA)
Anti-mouse IgG (Horseradish peroxidase): 115-035-003	G	1/20,000 of the stock solution (0.4 mg/ml)		Jackson Immunoresearch (West Grove, PA)
Anti-mouse IgG (Alexa Fluor 350, 546): A-11045, A-11030	G		1/2,000 of the stock solution (2 mg/ml)	Thermo Fisher Scientific (Waltham, MA)
Anti-rabbit IgG (Horseradish peroxidase): 5220-0336	G	1/40,000 of the stock solution (1 mg/ml)		Sera Care Life Sciences (Milford, MA)
Pregnant mare serum gonadotropin (PMG)			5 IU for superovulation	Aska Pharmaceutical Co. Ltd (Tokyo, Japan)
Human chrorinic gonadotropin (hCG)			5 IU for super ovulation	Aska Pharmaceutical Co. Ltd (Tokyo, Japan)

1) D, Donkey; G, Goat; M, Moues; R, Rabbbit. 2) IF: immunofluorescence, IEM: immunoelectron microscopy, IP: immunoprecipitation.

2) Iida H, et al, Tektin 4 is located on outer dense fibers, not associated with axonemal tubulins of flagella in rodent spermatozoa. *Mol Reprod Dev.* **73**, 929-936. doi: 10.1002/mrd.20486.PMID: 16596631 (2006).

Table S2. Primer sets used for PCR.

Name of primer pair	Sequence
Primer set for detecting mutation	5'-CCCCATTCTTGCTCAGACACTA-3'
	5'-CATCCTGGTTCACACCTCGC-3'
Primer set for sequencing	5'-CAGGAAACAGCTATGAC-3'
	5'GTTTTCCCAGTCACGAC-3'
Primer set for *Odf4* cDNA	5'-CCCCATTCTTGCTCAGACACTA-3'
	5'-TGCTCCTCCTTGGTCACGATG-3'
Primer set for *Odf2* cDNA	5'-ATGAAGGACCGATCTTCAACTCCC-3'
	5'ATAGGCAGGGGGCGATCGGGAGCG-3'
Primer set A for *Odf4-Egfp* genotyping	5'-CATCAGCGCTACCCCCAAAATGTCA-3'
	5'- GATGAACTTCAGGGTCAG-3'
Primer set B for *Odf2-mCherry* genotyping	5'-CCCCTATGGAGGACAAGCTCAACCAG-3'
	5'-CTTGTACAGCTCGTCCATGCCGC-3'
Primer set for *Odf4* RT-PCR	5'-CATCAGCGCTACCCCCAAAATGTCA-3'
	5'-GGTGGGACAAAACCCAGCCCTATG-3'
Primer set for *Gapdh* RT-PCR	5'-ACCACAGTCCATGCCATCAC-3'
	5'-TCCACCACCCTGTTGCTGTA-3'

Supplementary Fig. S1

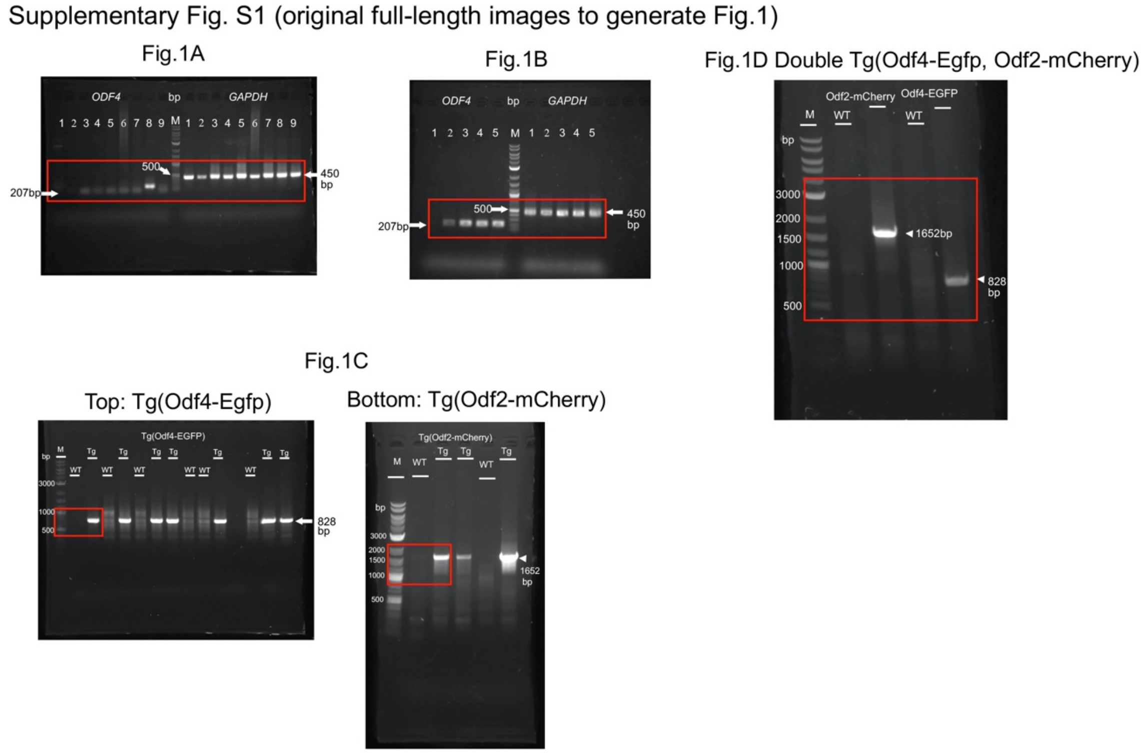

Fig. S1. Original full-length images to generate Fig. 1. (**A**) (**B**) RT–PCR. (**C**) (**D**) Genomic PCR. Red boxes denote the regions are shown in Fig 1. Explanations are written in Figure 1 legend.

Supplementary Fig. S2

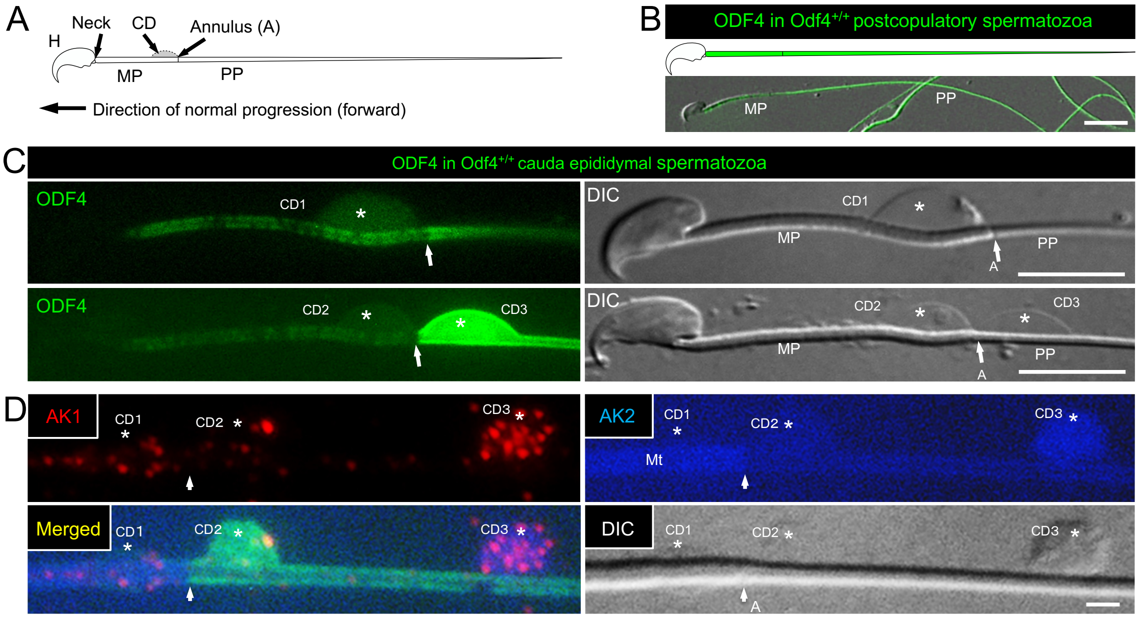

E

Protein	Genotype	Midpiece: Plasma membrane	Midpiece: Cytoplasmic droplet (CD)	Midpiece: Mitochondria (Mt)	Midpiece: ODF	Principal piece: Plasma membrane	Principal piece: CD (occasionally)	Principal piece: ODF
AK1	+/+	- (absent)	+ (present)	+/-	+ (weak)	-	+	+ (strong)
AK2	+/+	-	+	+ (strong)	-	-	+ (weak)	-
ODF4	+/+	+	+	+	+	+	+	+

Fig. S2. Localization of ODF4, AK1 and AK2 in wild-type spermatozoa. (**A**) Sperm regions: drawn by Toshimori. (**B – D**) Tg(Odf4-Egfp) spermatozoa. (**B**) Postcopulatory spermatozoa recovered from the uterine cavity 2 hours after copulation. An ODF4 green signal is observed in the whole flagellum. (**C**) and (**D**) Cauda epididymal spermatozoa. (**C**) Cytoplasmic droplet (CD; CD1-3), showing varied fluorescence strength. (**D**) CDs are double positive for antibodies against AK1 and AK2 in ODF4-EGFP. Three CDs (CD1, 2, 3: *) are double positive for antibodies against AK1 (red: Alexa 546) and AK2 (blue; Alexa 350) in the ODF4-EGFP signal (green). (**E**) Summary of fluorescence signals of ODF4, AK1, and AK2 based on the data from (**B – D)** and Fig. S3. A: annulus. DIC: differential interference contrast. H: head. MP: midpiece. Mt: mitochondria. PP: principal piece. Scale bars = 10 µm (**B**), 5 µm (**C**), 2 µm (**D**), shown in the DIC image of each set.

Supplementary Fig. S3

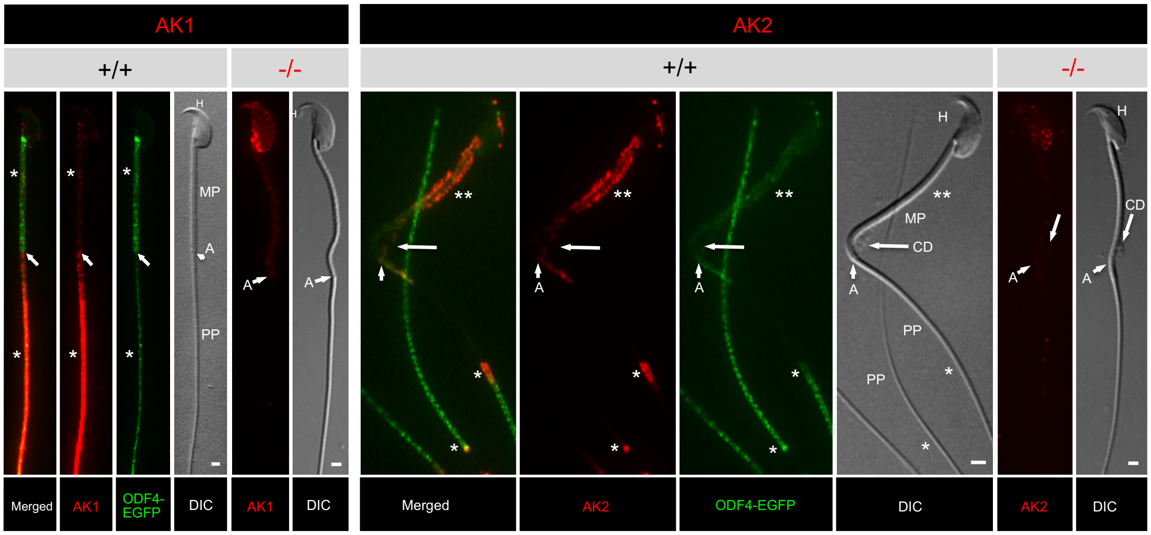

Fig. S3. Localization comparison of AK1 and AK2 with ODF4. +/+: *Odf4*$^{+/+}$. -/-: *Odf4*$^{-/-}$. Immunofluorescence with antibodies against AK1 and AK2 in Tg(Odf4-Egfp) spermatozoa. Green: ODF4-EGFP. Red (Alexa 546) indicates AK1 or AK2. AK1 (*Left*) +/+: The fluorescence signal is substantially detected in the whole flagellum. The midpiece is rather weakly stained, showing a patchy pattern presumably due to the physical barrier of tightly packed mitochondria (**). -/-: No red signals. AK2 (*Right*) +/+: AK2 is detected in the whole flagellum with a strong staining pattern in the midpiece (**) and with a scattered pattern in the principal piece (*). -/-: No red signals are observed. A: annulus. DIC: differential interference contrast. H: head. MP: midpiece. PP: principal piece. Scale bars = 2 µm, shown in the DIC image of each set.

Supplementary Fig. S4

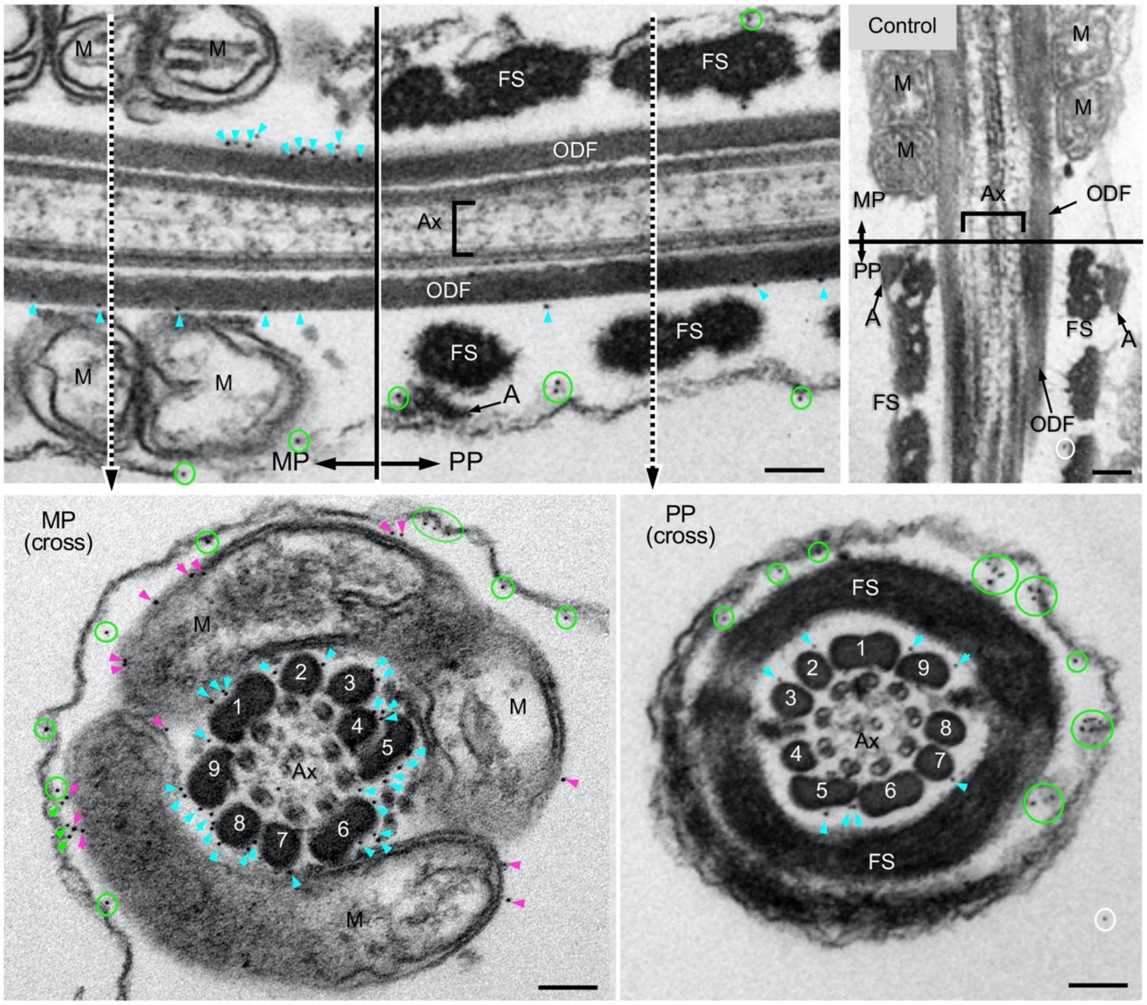

Fig. S4. ODF4 localization shown by immunogold electron microscopy. An antibody conjugated with 5-nm gold particles against EGFP was applied to Tg(Odf4-Egfp) spermatozoa. Immunogold particles are localized to the plasmalemma at the subplasmalemmal region (green circles) and 1–9 ODFs (cyan arrowheads) throughout the flagellum and around the mitochondria (magenta arrowheads) in the midpiece. No immunogold particles are observed in the control *Odf4*$^{+/+}$ (upper *right*), except for a few background particles indicated by a white circle at the lower right corner of the principal piece cross section. A: annulus. Ax: axoneme. FS: fibrous sheath. H: head. M: mitochondria. MP: midpiece. ODF: outer dense fiber. PP: principal piece. Scale bar = 100 nm.

Supplementary Fig. S5

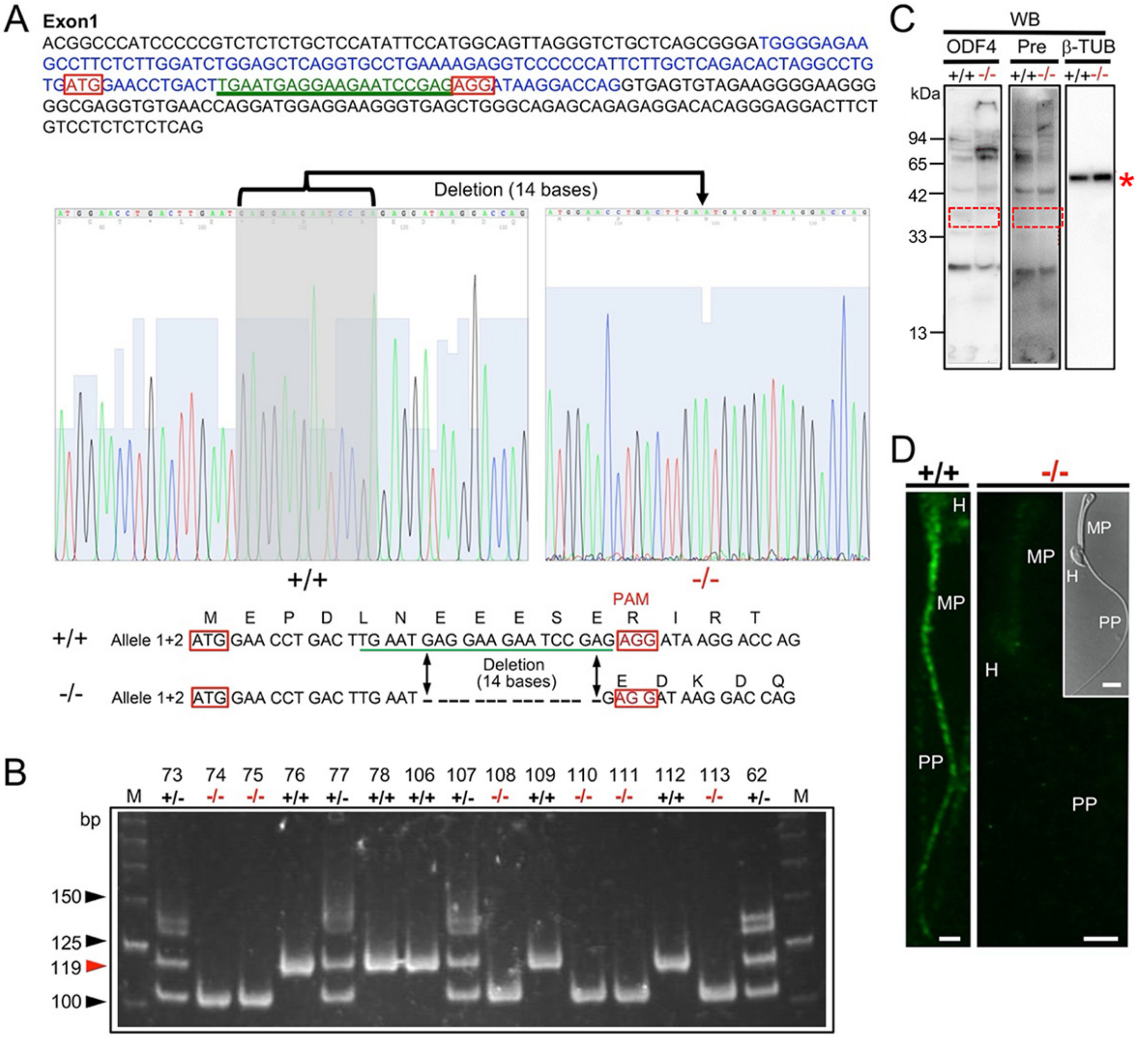

Fig. S5. Generation of *Odf4*$^{-/-}$ mice. +/+: Wild-type. *Odf4*$^{+/+}$. +/-: Heterozygous *Odf4*$^{+/-}$. -/-: Homozygous *Odf4*$^{-/-}$. (**A**) Gene sequence. (*Top*) Exon 1 sequence (blue). Underlined capital letters are the target sequence of the guide RNA. ATG: start codon. AGG: PAM (protospacer adjacent motif). (*Bottom*) Fourteen bases (GAGGAAGAATCCGA) were deleted in the generated *Odf4*$^{-/-}$ mice. (**B**) Genotype PCR using acrylamide gel (original image). Numerals at the top of the panel indicate the serial number of mutant mice. An *Odf4* exon 1 band with 119 base pairs (bp) is missing in *Odf4*$^{-/-}$ mice (red arrowhead). M: Markers for 150, 125, 100 (bp). (**C**) Cropped blots with black outlines for ODF4 antibody. Preimmune (Pre) and β-TUBULIN (β-TUB) antibody are from the same membrane. Original full-length images with different exposure times are shown in the supplementary SD1 for anti ODF4 antibody and Preimmune. Further enlarged single file is shown in SD17 for SD1E (ODF4), SD18 for SD1F (β-TUBULIN) and SD19 for SD1J (Preimmune). Western blotting using an antibody against ODF4 (Bios). The rabbit polyclonal antibody against ODF4 was custom-made by raising against a synthetic peptide (C-ENSQESPKDDQKPS) corresponding to residues 262–275 conjugated to keyhole limpet hemocyanin. ODF4 bands (approximately 35 kDa: red rectangles) are not detected in *Odf4*$^{-/-}$ mice (ODF4) or by a preimmune antibody (Pre). Control: β-TUBULIN (*: β-TUB) is present in *Odf4*$^{-/-}$

and *Odf4*$^{+/+}$. (**D**) Immunofluorescence using the custom-made antibody against ODF4. The signal is detected in *Odf4*$^{+/+}$ spermatozoa (*Left*) but not in *Odf4*$^{-/-}$ (*Right*) spermatozoa, except as background signals (*Inset*: DIC). Scale bar = 10 µm (+/+), 5 µm (-/-).

Supplementary Fig. S6

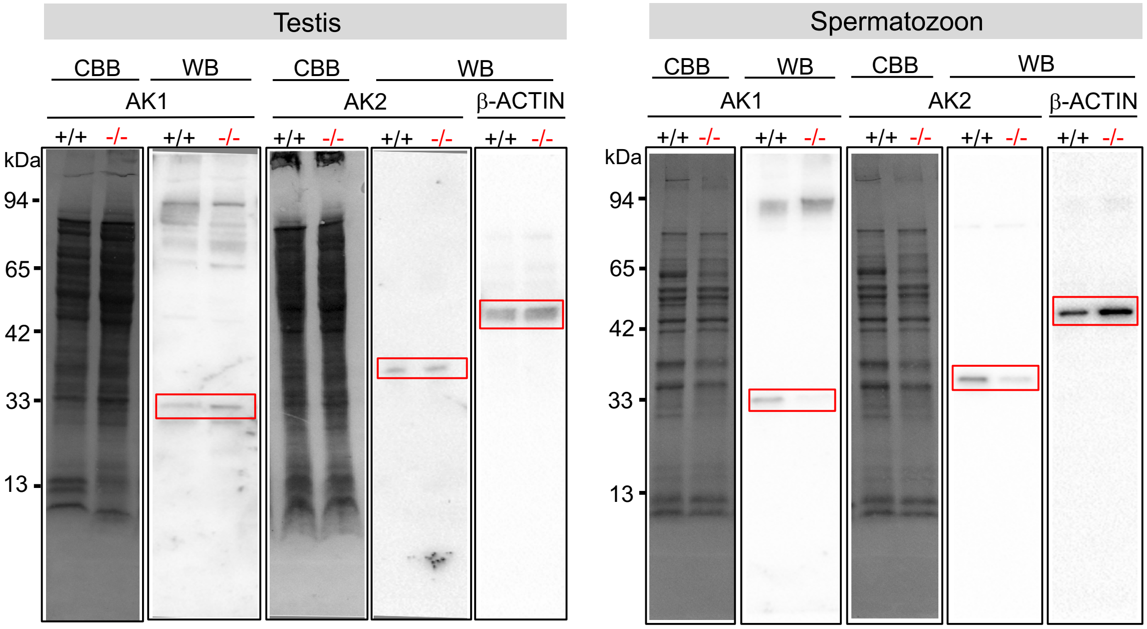

Fig. S6. AK1 and AK2 images for Fig. 4A left (Testis and Spermatozoa). Cropped images for Fig. 4A left (testis and spermatozoon). Coomassie Brilliant Blue (CBB) and western blotting. +/+: *Odf4*$^{+/+}$. -/-: *Odf4*$^{-/-}$. β-ACTIN (42 kDa): internal control (*). Original full-length images are shown in the supplementary SD2 (testis) and SD3 (spermatozoon). Further enlarged single file is shown in SD20 for SD2F (Testis AK1), SD21 for SD2K (Testis AK2), SD22 for SD2L (Testis β-ACTIN) and SD23 for SD2M (CBB). Also, further enlarged single file is shown in SD24 for SD3F (Sperm AK1 and AK2), SD25 for SD3G (Sperm β-ACTIN, and SD26 for SD3H (CBB). Each set of proteins was detected on the same membrane, and the same amount per lane (30 μg for testis samples and 15 μg for sperm samples) was loaded as described below. (*Left*) Testis. AK1 (23 kDa) and AK2 (30 kDa) are positive in *Odf4*$^{-/-}$ samples (rectangles). (*Right*) Sperm. Both AK1 and AK2 are positive in *Odf4*$^{+/+}$ mice but significantly reduced in *Odf4*$^{-/-}$ mice (rectangles).

Supplementary Fig. S7

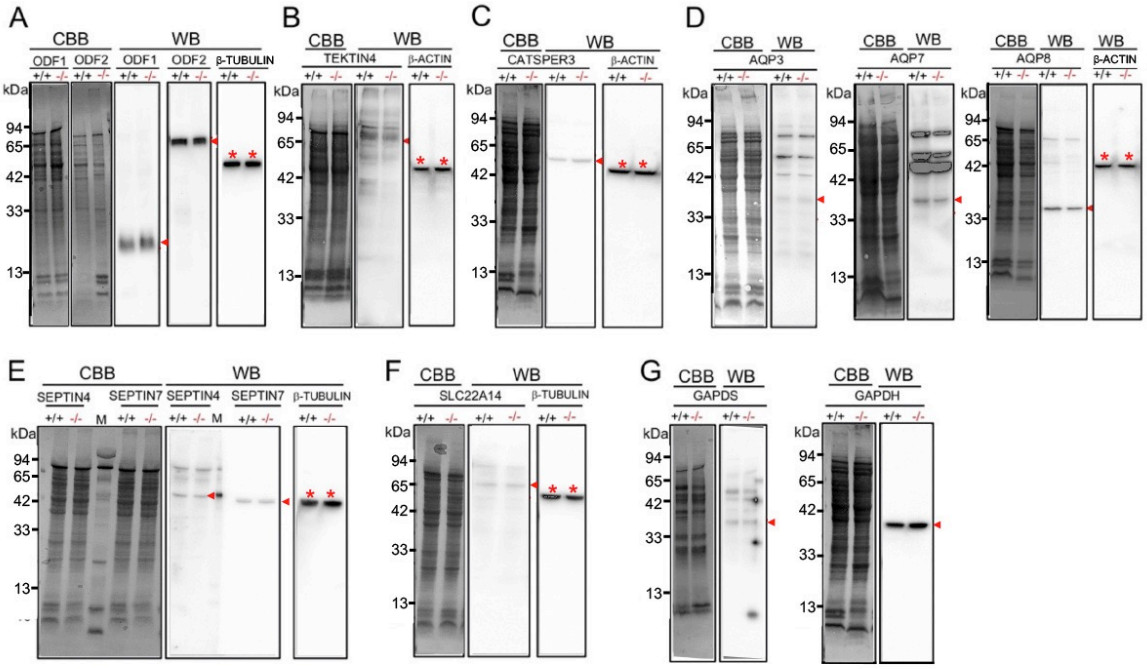

Fig. S7. CBB and western blotting images of flagellar proteins for Fig. 4A right. (**A** – **G**) Western blotting. Cropped images from the different parts of the different gel. Original full-length images with different exposure times are shown in the supplementary SD4 for S7A, SD5 for S7B, SD6 for S7C, SD7 for S7D *left*, SD8 for S7D *middle*, SD9 for S7D *right*, SD10 for S7E, SD11 for S7F, and SD12 for S7G *left*, and SD13 for S7G *right*. **A**. Further enlarged single file is shown in SD29 for SD4F (Sperm ODF2 and ODF1), SD30 for SD4G (Sperm β-TUBULIN), and SD31 for SD4H (CBB). **B**. Further enlarged single file is shown in SD32 for SD5E (Sperm TEKTIN4); SD33 for SD5F (Sperm β-ACTIN), and SD34 for SD5G (CBB). **C**. Further enlarged single file is shown in SD35 for SD6A (Sperm CATSPER3 and β-ACTIN), and SD36 for SD6E (CBB). **D**. Further enlarged single file is shown in SD37 for SD7E (Sperm AQP3), and SD38 for SD7F (CBB); SD39 for SD8E (Sperm AQP7), and SD40 for SD8F (CBB); SD41 for SD9B (Sperm AQP8), SD42 for SD9E (CBB), and SD43 for SD9F (Sperm β-ACTIN). **E**. Further enlarged single file is shown in SD44 for SD10A (Sperm SEPTIN4 and SEPTIN7), SD45 for SD10E (CBB), and SD46 for SD10F (Sperm β-TUBULIN). **F**. Further enlarged single file is shown in SD47 for SD11F (Sperm SLC22A), SD48 for SD11G (CBB), and SD49 for SD11H (Sperm β-TUBULIN). **G**. Further enlarged single file is shown in SD50 for SD12C (Sperm GAPDS), and SD51 for SD12F (CBB); SD52 for SD13A (Sperm GAPDH), and SD53 for SD13D (CBB). The same amount of protein was loaded per lane after extraction by RIPA or urea solutions, as written in brackets for each experiment (below). Expected bands (kDa) are indicated by arrowheads in each experiment. (**A**) ODF1 (27–30 kDa: 10 μg, urea) and ODF2 (70 kDa: 5 μg, urea). (**B**) TEKTIN4 (50 kDa: 20 μg, urea). (**C**) CATSPER3 (46 kDa: 30 μg, RIPA). (**D**) AQP3 (32 kDa: 20 μg, RIPA), AQP7 (23–33 kDa: 50 μg, RIPA) and AQP8 (30–32 kDa: 30 μg, RIPA). (**E**) SEPTIN 4 (50 kDa: 20 μg, RIPA) and SEPTIN 7 (48–50 kDa: 20 μg, RIPA). (**F**)

SLC22A14 (62 kDa: 20 μg, RIPA). (**G**) GAPDS (40–45 kDa: 25 μg, RIPA) and GAPDH (34 kDa: 25 μg, RIPA). *: control β-ACTIN (44 kDa: 20–50 μg, RIPA or urea depending on the experimental purpose) and β-TUBULIN (55 kDa: 5–10 μg, RIPA or urea depending on the experimental purpose). M: Marker.

Supplementary Fig. S8

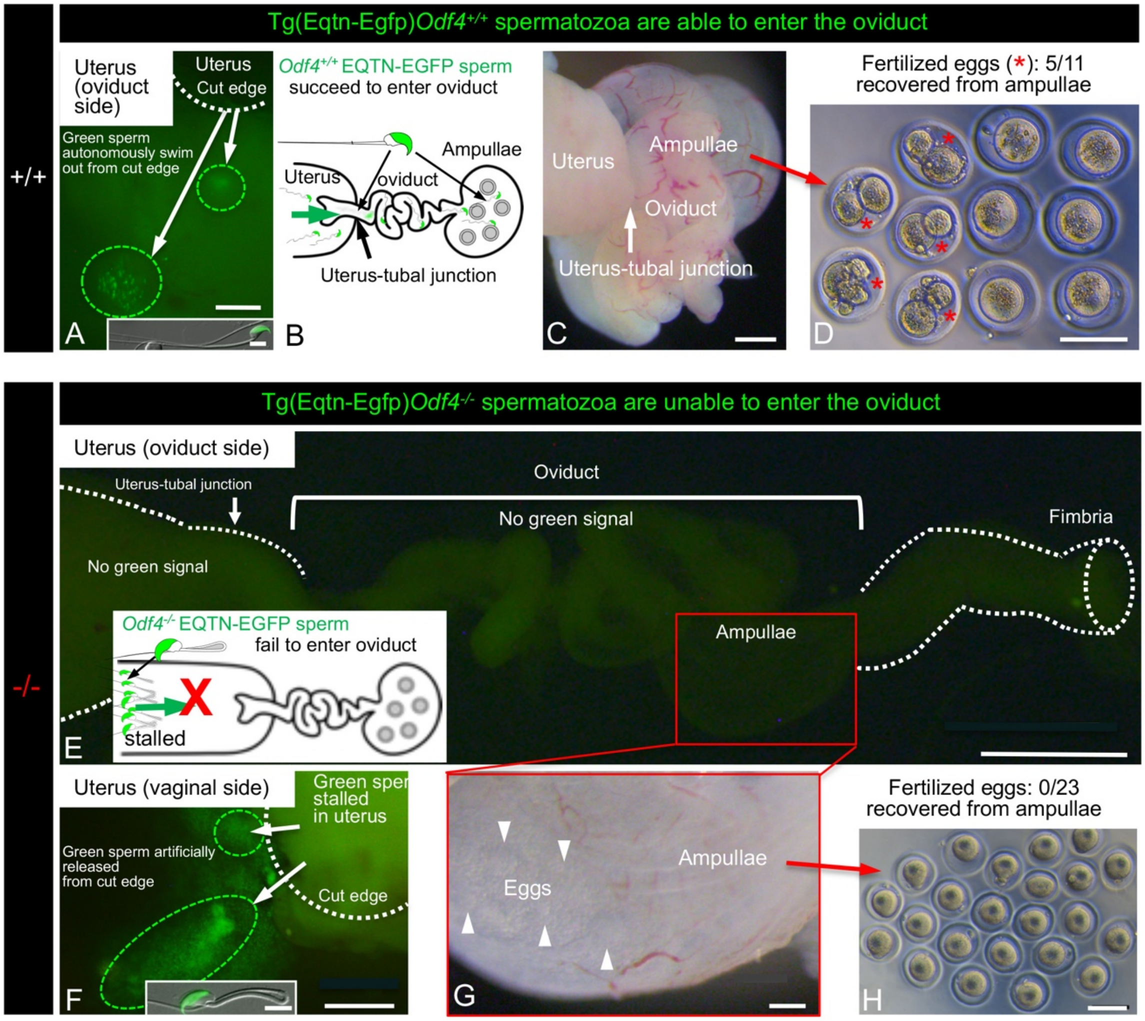

Fig. S8. Sperm ascension in the female reproductive tract. +/+: *Odf4*$^{+/+}$ (Control). -/-: *Odf4*$^{-/-}$. The purpose of this experiment was to determine how *Odf4*$^{-/-}$ spermatozoa behave and ascend in the *Odf4*$^{+/+}$ female reproductive tract compared to control *Odf4*$^{+/+}$ sperm behavior and ascension. Pregnant mare serum gonadotropin (PMSG) and human chorionic gonadotropin (hCG) (ASKA Pharmaceutical Co., Ltd.), 5 IU each, were used to induce superovulation in female mice. The males (spermatozoa) used were Tg(Eqtn-Egfp)*Odf4*$^{+/+}$ (control; +/+) and Tg(Eqtn-Egfp)*Odf4*$^{-/-}$ (-/-). (**A – D**) *Odf4*$^{+/+}$ Tg spermatozoa can ascend in the female reproductive tract without any trouble (**A**), enter the oviduct (**B, C**), and fertilize oocytes; here, 5 two-cell zygotes and 6 one-cell oocytes recovered from one side of ampullae are shown (**D**). (**E – H**) *Odf4*$^{-/-}$ spermatozoa stall in the lower region (vaginal side) of the uterus (**E**, **F**). No *Odf4*$^{-/-}$ Tg spermatozoa were found in any region of the upper part (oviduct side) of the uterus throughout the ampulla (**E**), and fertilization did not occur; all 23 eggs recovered from both sides of the ampullas were unfertilized and were one-cell oocytes (**G**, **H**). Eggs recovered approximately 3 hours after plug formation. Green Tg spermatozoa used in this experiment are released from the cut edge (circles), as shown in insets of **A** (*Odf4*$^{+/+}$ Tg) and **F** (*Odf4*$^{-/-}$ Tg); green heads (acrosomes) that express EGFP are shown. Experiments were duplicated using different pairs to confirm the result. Scale bars = 0.5 mm (**A**, **C**, **F**), 1 mm (**E**), 5 µm (*Inset*; **A**, **F**), 100 µm (**D**, **G**, **H**). **B**, **E**: drawn by Toshimori.

Supplementary Fig. S9

A

*N*trials	+/+		-/-	
	Capacitation			
	Before	After	Before	After
1	4.0[1)]	3.0	23.2	7.0
2	3.9	3.0	23.1	7.2
3	2.7	3.2	15.8	9.8
4	2.6	3.1	15.6	10.1
5	2.9	2.0	48.3	8.8
6	2.6	2.2	46.0	7.6
7	3.6	3.5	34.6	5.8
8	3.5	4.0	29.0	5.0
9	2.3	2.2	-	-
Average ± SEM	3.1 ± 4.5	2.9 ± 0.2	29.4 ± 4.5	7.7 ± 0.6

1) nmole/1x10^4 sperm.

B

*N*trials	+/+		-/-	
	Capacitation			
	Before	After	Before	After
1	1,119.7[1)]	1,292.7	2,344.7	2,186.2
2	1,580	2,135	2,470.7	2,599
3	1,159	658	4,919	1,873
4	1,024	761	4,835	1,542
5	931	778	3,592	1,905
6	1,063	775	3,816	1,913
Average ± SEM	1,146.1 ± 92.6	1,066.6 ± 232.5	3,662.9 ± 452.4	2,003.0 ± 145.6

1) Read luminescence (RLU)/1x10^4 sperm.

Fig. S9. Raw data of average ATP and ADP concentrations in spermatozoa before and after capacitation. +/+: *Odf4*$^{+/+}$. -/-: *Odf4*$^{-/-}$. (**A**) ATP concentration (nmol/1x10^4 sperm). *N*trials: 9 trials from 4 different males for *Odf4*$^{+/+}$ and *Odf4*-null mice. (**B**) ADP concentration (RLU/1x10^4 sperm). *N*trials: 6 trials from 6 different males for *Odf4*$^{+/+}$ and *Odf4*-null mice.

Supplementary Fig. S10

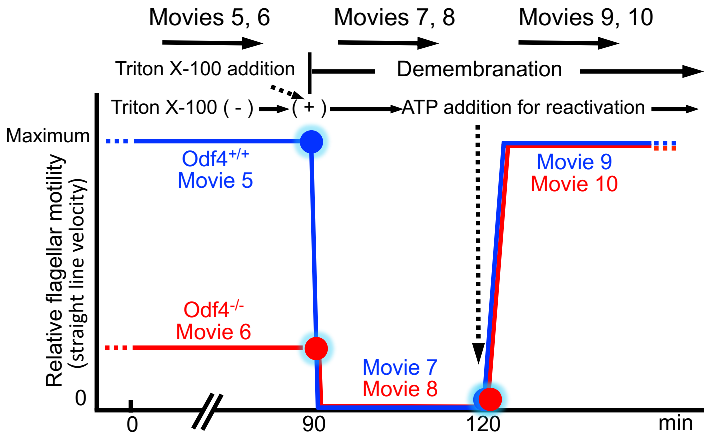

Genotype (Movies)	Demembranation (Triton X-100 treatment)	Cytoplasmic droplet	Flagellar shape	ATP addition for reactivation: No	ATP addition for reactivation: Yes
				Sperm motility (straight line velocity) and progression	
+/+ (Movies 5,7,9)	Before	absent or present → absent (naturally)	Straight	Forward	–
	After	absent 1)	Straight	– (immotile)	Forward (reactivated)
–/– (Movies 6,8,10)	Before	present	Hairpin	Backward	–
	After	absent 1)	Straight	– (immotile)	Forward (reactivated)

1) Cytoplasmic droplets are lost by Triton X-100 treatment.

Fig. S10. Demembranation assay. +/+: *Odf4*$^{+/+}$. -/-: *Odf4*$^{-/-}$. (*Top*) Diagram showing the experimental design and video analysis (blue line for +/+, red line for -/-). See the methods described above for the preparation and recording of spermatozoa. For this assay, we evaluated flagellar motility (relative straight-line velocity) because the purpose of this study was to determine whether demembranated spermatozoa can resume movement. Analyses were performed only by visual observation, and these results were not statistically examined because statistical analysis for the videos was difficult because individual spermatozoa in each experiment resumed movement in various ways and at various times, depending on the positions where the spermatozoa were localized, especially during the assay after ATP addition (reactivation assay). Here, we show the most representative image for each experiment (Videos S5 - S10). All results are written in the main text for the demembranation assay and are summarized in a table in this figure (*Bottom*). Ordinate: relative motility (frequency and forward motility observed by eye). Abscissa: time (minutes) after the start of the experiment.

Supplementary Fig. S11

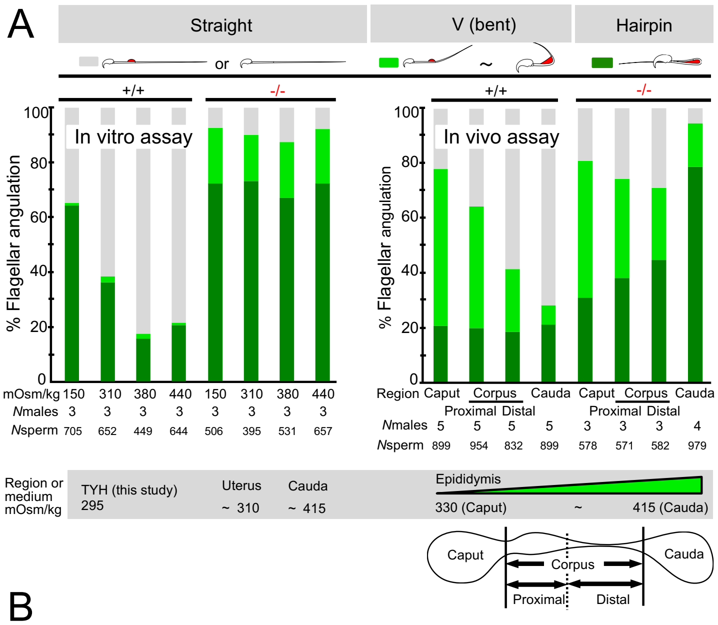

B

	+/+					-/-				
Osmolarity (mOsm/kg)	150	310	380	440	Total	150	310	380	440	Total
Straight [1)]	245 [2)]	401	370	505	1,521	38	39	67	51	195
V [1)]	7	15	8	8	38	103	68	109	132	412
Hairpin [1)]	453	236	71	131	891	365	288	355	474	1,482
Total	705	652	449	644	2,450	506	395	531	657	2,089
Straight (%)	34.8	61.5	82.4	78.4	-	7.5	9.9	12.6	7.8	-
V (%)	1.0	2.3	1.8	1.2	-	20.4	17.2	20.5	20.1	-
Hairpin (%)	64.3	36.2	15.8	20.3	-	72.1	72.9	66.9	72.2	-
Total (%)	100	100	100	100	-	100	100	100	100	-

1) Sperm flagellar morphology. 2) *N*sperm.

	+/+					-/-				
Epididymis	Caput	Corpus		Cauda	Total	Caput	Corpus		Cauda	Total
		Proximal	Distal				Proximal	Distal		
Straight [1)]	198 [2)]	341	486	644	1,669	110	146	167	55	478
V [1)]	512	421	190	61	1,184	287	205	154	153	799
Hairpin [1)]	189	192	156	194	731	181	220	261	771	1,433
Total	899	954	832	899	3,584	578	571	582	979	2,710
Straight (%)	22.0	35.7	58.4	71.6	-	19.0	25.4	28.7	5.6	-
V (%)	57.0	44.1	22.8	6.8	-	49.7	35.7	26.5	15.6	-
Hairpin (%)	21.0	20.1	18.8	21.6	-	31.3	38.3	44.9	78.8	-
Total (%)	100	100	100	100	-	100	100	100	100	-

1) Sperm flagellar morphology. 2) *N*sperm.

Fig. S11. Effect of environmental osmolarity changes on *Odf4*$^{-/-}$ flagellar angulation. These experiments were performed in TYH culture medium (in vitro assay: **A**, *left*) and during sperm passage through the epididymis (in vivo assay: **A,** *right*). The result is shown as percent (%) sperm flagellar angulation (angulation rate). +/+: *Odf4*$^{+/+}$. -/-: *Odf4*$^{-/-}$. Criteria for flagellar shape are shown at the top of the panel. Gray bars indicate straight shapes, light green bars indicate V shapes, and dark green bars indicate hairpin shapes. (**A**) % flagellar angulation. (*Left*) Control *Odf4*$^{+/+}$ spermatozoa (+/+). The angulation rate gradually decreases, while in *Odf4*$^{-/-}$ spermatozoa (-/-), it is always high at approximately 90%. *N*males/*N*sperm: 3/2,450 for *Odf4*$^{+/+}$ and 3/2,089 for *Odf4*$^{-/-}$. (*Right*) In the *Odf4*$^{+/+}$ spermatozoa (+/+), the angulation rate (%) of V (bent) plus hairpin sperm flagella decreases, where the percentage (%) of hairpin sperm flagella is constant at approximately 20%; in contrast, in *Odf4*$^{-/-}$ spermatozoa (-/-), it is always high at approximately 70–98%, with an increasing rate of hairpin flagellum from 30% to 80%. *N*males/*N*sperm; 5/3,584 for *Odf4*$^{++}$, and 4/2,710 for *Odf4*$^{-/-}$. (**B**) Raw data for **A**. *Top* for **A**, *Left*. (*Bottom*) Raw data for **A**, *right*.

Supplementary Fig. S12

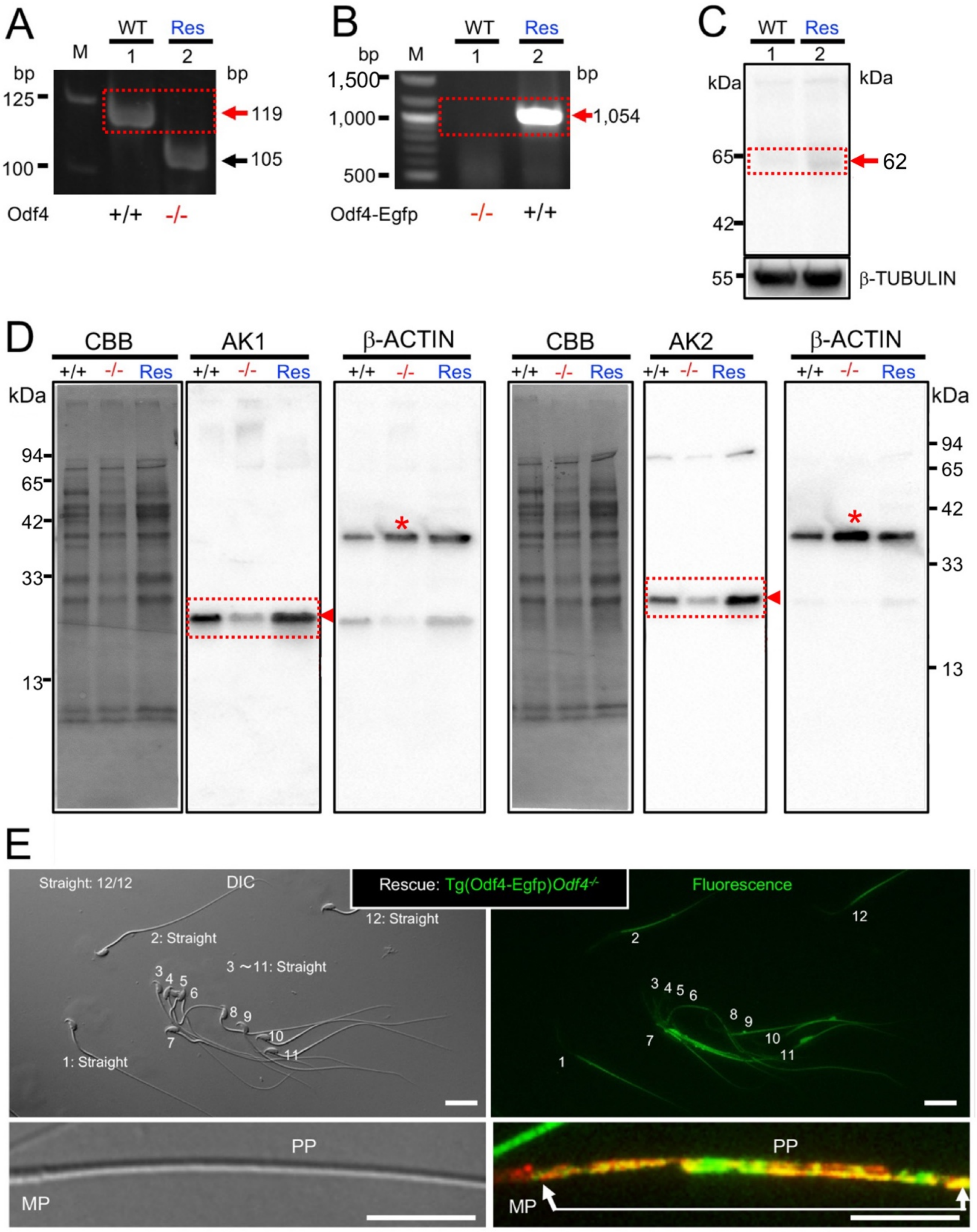

Fig. S12. Rescue experiment. WT or +/+: *Odf4*$^{+/+}$. -/-: *Odf4*$^{-/-}$. Rescue or Res; Tg(Odf4-Egfp)*Odf4*$^{-/-}$. (**A** – **C**) Production of the rescue mice. Lane 1: WT. Lane 2: Rescue. Sperm extracts for Lanes 1 and 2 in a and b are from the same males. (**A**) Genomic PCR using an acrylamide gel

(original image). The *Odf4* exon 1 band (119 base pairs: bp) (red arrowhead) is absent in the rescue group (Lane 2). (**B**) Genomic PCR using an agarose gel (original image). The band for the *Odf4-EGFP* gene (1,054 bp) (red arrowhead) is present only in the rescue (Lane 2). (**C**) Western blotting with an antibody against GFP. A 62 kDa band (29 kDa EGFP + 33 kDa ODF4) (red arrowhead) is present in the rescue (Lane 2). β-ACTIN: Control. Original full-length images with different exposure times are shown in the supplementary SD15. Further enlarged single file is shown in SD55 for SD15A, and SD56 for SD15D for the rescued mice. (**D**) Coomassie Brilliant Blue (CBB) and western blotting for sperm extracts. Western blotting with antibodies against AK1 (*Left*) and AK2 *(Right*) shows corresponding bands in the rescue (rectangles indicated by arrows); this experiment with the control β-ACTIN (*) was performed on the same membrane. Original full-length images with different exposure times are shown in the supplementary SD16. Further enlarged single file is shown in SD57 for SD16E, SD58 for SD16F, and SD57 for CBB. (**E**) Fluorescence images of the rescued spermatozoa. (*Top*) All rescued sperm flagella (green) are straight, as shown by DIC (*Left*) and fluorescence (*Right*). (*Bottom*) Immunofluorescence using a custom-made antibody against ODF4 labeled with Alexa 546. The rescued spermatozoa treated with this antibody (red signal) after detergent (saponin) treatment show yellow color images (between arrows) owing to overlapping ODF4-EGFP (green) and Alexa-546-labeled ODF (red) colors when observing the well-exposed regions in whole flagella. Scale bars = 10 μm.
