# Supplementary material for: The association of ODF4 with AK1 and AK2 in mice is essential for fertility through its contribution to flagellar shape
Source: Sci Rep. 2023 Feb 20;13:2969. doi: 10.1038/s41598-023-28177-z (PMC9941515; doi:10.1038/s41598-023-28177-z)
Supplement: Supplementary file 2 — Supplementary Information 2. [file 41598_2023_28177_MOESM2_ESM.pdf]

Supplementary Dataset (SD)

SD1- SD57: Original figures used in the main manuscript and Supplementary Information (SI). Among them, **SD1** - **SD16** contains the files of combined figures with different exposure times, and **SD17** -**SD57** contains the further enlarged single file of each figure selected in **SD1** – **SD16** for the main manuscript and SI. +/+; *Odf4*$^{+/+}$. -/-; *Odf4*$^{-/-}$. RES; Rescue. M; marker proteins (protein molecular marker and/or prestained marker). The signals were analyzed by Image Lab Software from the Molecular Imager ChemiDoc XRS Plus (Bio-Rad).

SD1: Original data for Fig S5C ODF4
This file contains the full-length images with different exposure times. Red box regions with dotted line boxes in **E** and **J** are used for Fig S5C for anti ODF4 antibody and preimmune, respectively. Red box region in **F** is used for the control internal protein (β-TUBULIN). The membrane for the western blotting with an antibody against ODF4 is different from that for preimmune and β-TUBULIN which are on the same membrane. Further enlarged single file is shown in **SD17** for **SD1E** (ODF4), **SD18** for **SD1F** (β-TUBULIN) and **SD19** for **SD1J** (Preimmune).

SD2: Original data for Fig S6 AK1 and AK2 to generate Fig 4A left top (Testis)
This file contains the full-length images with different exposure times (same membrane), excepting the control internal protein (β-ACTIN). Red box regions in **F** and **K** are used for Fig S6 AK1 and AK2, respectively. Red box region in **L** is used for the control internal protein (β-ACTIN). Red box regions in **M** (Coomassie Brilliant Blue: CBB) are used for the western blotting of AK1 and AK2. Further enlarged single file is shown in **SD20** for **SD2F** (Testis AK1), **SD21** for **SD2K** (Testis AK2), **SD22** for **SD2L** (Testis β-ACTIN) and **SD23** for **SD2M** (CBB).

SD3: Original data for Fig S6 AK1 and AK2 to generate Fig 4A left bottom (Spermatozoon). This file contains the full-length images with different exposure times. Red box regions indicated by solid line in **F** and **H** (CBB) are used for Fig S6 AK1 and AK2. Red box regions indicated by dotted line in **F** and **H** (CBB) are used for Fig 4A left (bottom) AK1 and AK2. Red box regions indicated by solid line and dotted line in **G** are used for Fig S6 and Fig 4A left (bottom) control internal protein (β-ACTIN), respectively. Red box regions in **J** (anti-ODF4 antibody) and **K** (anti β-TUBULIN) are used for Fig 4A (bottom). The membrane for AK1, AK2 and β-ACTIN is same, which is different from the membrane for ODF4 and the control internal protein β-ACTIN. Further enlarged single file is shown in **SD24** for **SD3F** (Sperm AK1 and AK2), **SD25** for **SD3G** (Sperm β-ACTIN, **SD26** for **SD3H** (CBB), and **SD27** for **SD3J** (Sperm ODF4).

SD4: Original data for Fig S7A ODF1 and ODF2 to generate Fig 4A right (Spermatozoon). This file contains the full-length images with different exposure times

for Fig S7A. Red box regions in **F** are used for Fig S7A. The order of anti-ODF2 and anti-ODF1 from the left side in SD4 is rearranged as ODF1 and ODF2 from the left side in Fig S7A. Red box region in **G** is used for the control internal protein (β-TUBULIN) in Fig S7A. Red box regions in **H** (CBB) are used for the ODF1 and ODF2 western blotting. The membrane is same for ODF1, ODF2 and β-TUBULIN. Further enlarged single file is shown in **SD29** for **SD4F** (Sperm ODF2 and ODF1), **SD30** for **SD4G** (Sperm β-TUBULIN), and **SD31** for **SD4H** (CBB).

SD5: Original data for Fig S7B TEKTIN4 to generate Fig 4A right (Spermatozoon). This file contains the full-length images with different exposure times. Red box regions in **E** and **G** (CBB) are used for Fig S7B TEKTIN4. Red box region in **F** is used for the control internal protein (β-ACTIN). The membrane for TEKTIN4 is different from that for β-ACTIN. Further enlarged single file is shown in **SD32** for **SD5E** (Sperm TEKTIN4), **SD33** for **SD5F** (Sperm β-ACTIN), and **SD34** for **SD5G** (CBB).

SD6: Original data for Fig S7C CATSPER3 to generate Fig 4A right (Spermatozoon). This file contains the full-length images with different exposure times. Red box regions in **A** are used for Fig S7C CATSPER3 and the control internal protein (β-ACTIN). Red box region in **E** (CBB) is used for Fig S7C CATSPER3. The membrane for CATSPER3 and β-ACTIN is same. Further enlarged single file is shown in **SD35** for **SD6A** (Sperm CATSPER3 and β-ACTIN), and **SD36** for **SD6E** (CBB).

SD7: Original data for Fig S7D AQP3 to generate Fig 4A right (Spermatozoon). This file contains the full-length images with different exposure times. Red box regions in **E** and **F** (CBB) are used for Fig S7D AQP3. Further enlarged single file is shown in **SD37** for **SD7E** (Sperm AQP3), and **SD38** for **SD7F** (CBB).

SD8: Original data for Fig S7D AQP7 to generate Fig 4A right (Spermatozoon). This file contains the full-length images with different exposure times. Red box regions in **E** and **F** (CBB) are used for Fig S7D AQP7. Further enlarged single file is shown in **SD39** for **SD8E** (Sperm AQP7), and **SD40** for **SD8F** (CBB).

SD9: Original data for Fig S7D AQP8 to generate Fig 4A right (Spermatozoon). This file contains the full-length images with different exposure times. Red box regions in **B** and **E** (CBB) are used for Fig S7D AQP8. Red box region in **F** is used for the control internal protein (β-ACTIN). Further enlarged single file is shown in **SD41** for **SD9B** (Sperm AQP8), **SD42** for **SD9E** (CBB), and **SD43** for **SD9F** (Sperm β-ACTIN).

SD10: Original data for Fig S7E SEPTIN4 and SEPTIN7 to generate Fig 4A right (Spermatozoon). This file contains the full-length images with different exposure times. Red box regions in **A** and **E** (CBB) are used for Fig S7E SEPTIN4 and SEPTIN7, respectively. Red box region in **F** is used for the control internal protein (β-TUBULIN). The membrane is same for SEPTIN4, SEPTIN7 and β-TUBULIN. Further

enlarged single file is shown in **SD44** for **SD10A** (Sperm SEPTIN4 and SEPTIN7), **SD45** for **SD10E** (CBB), and **SD46** for **SD10F** (Sperm β-TUBULIN).

SD11: Original data for Fig S7F SLC22A14 to generate Fig 4A right (Spermatozoon). This file contains the full-length images with different exposure times. Red box regions in **F** and **G** (CBB) are used for Fig S7F SLC22A14. Red box region in **H** is used for the control internal protein (β-TUBULIN). The membrane is same for SLC22A14 and β-TUBULIN. Further enlarged single file is shown in **SD47** for **SD11F** (Sperm SLC22A), **SD48** for **SD11G** (CBB), and **SD49** for **SD11H** (Sperm β-TUBULIN).

SD12: Original data for Fig S7G GAPDS to generate Fig 4A right (Spermatozoon). This file contains the full-length images with different exposure times. Red box regions in **C** and **F** (CBB) are used for Fig S7G GAPDS. Further enlarged single file is shown in **SD50** for **SD12C** (Sperm GAPDS), and **SD51** for **SD12F** (CBB).

SD13: Original data for Fig S7G GAPDH to generate Fig 4A right (Spermatozoon). This file contains the full-length images with different exposure times. Red box regions in **A** and **D** (CBB) are used for Fig S7G GAPDH. Further enlarged single file is shown in **SD52** for **SD13A** (Sperm GAPDH), and **SD53** for **SD13D** (CBB).

SD14: Original data for Fig 4B (IP). This file contains the full-length images with different exposure times (same membrane). Red box region in **E** is used for Fig 4B (IP). The membrane is same for AK1 and AK2. Further enlarged single file is shown in **SD54** for AK1 and AK2 of Tg and Odf4$^{-/-}$ (-/-), and the positive control AK1 and AK2.

SD15: Original data for Fig S12C Tg(Odf4-Egfp)*Odf4*$^{-/-}$ to generate Fig 7C (Rescue). This file contains the full-length images with different exposure times. Red box region indicated by solid line with red box indicated by dotted line for 62 kDa band level in **A** is used for Fig S12C (ODF4-EGFP protein recognized by anti-GFP antibody). Red box region in **D** is used for the control internal protein (β-TUBULIN). The membrane is same for GFP and β-TUBULIN. Further enlarged single file is shown in **SD55** for **SD15A** for the rescued mice (RES), showing the absence of the inserted GFP in the wild-type mice (WT) and the presence of the inserted GFP in the RES. Also, further enlarged single file is shown in **SD56** for **SD15D** for the rescued mice (RES), showing the internal control, β-TUBULIN for **SD55**.

SD16: **Original data for Fig S12D AK1 and AK2 to generate Fig 7C (Rescue).** This file contains the full-length images with different exposure times (same membrane). Red box regions indicated by solid line and red boxes indicated by dotted line in **E** (western blotting) and **G** (CBB) are used for Fig S12D and for Fig 7C (AK1 and AK2), respectively. Red box regions indicated by solid line and red boxes indicated by dotted line in **F** are used for the control internal protein (β-ACTIN) for Fig S12D and Fig 7C,

respectively. The membrane is same for AK1. AK2 and β-ACTIN. Further enlarged single file is shown in **SD57** for **SD16E** rescued study (RES), showing that both the signals of AK1 and AK2 in the rescued mice are strong as in the wild-type mice Odf4$^{+/+}$ (+/+). The signal is quite low in Odf4$^{-/-}$ (-/-). Further enlarged single file is shown in **SD58** for **SD16F** rescued study (RES), showing the control internal protein, β-TUBULIN. Further enlarged single file is shown in **SD59** for **SD16G** rescued study (RES), showing the corresponding CBB.

SD17-SD19: Single file for SD1.
SD17: for SD1E (ODF4).
SD18: for SD1F (β-TUBULIN).
SD19: for SD1J (Preimmune).

SD20-SD23: Single file for SD2.
SD20: for SD2F (Testis AK1).
SD21: for SD2K (Testis AK2).
SD22: for SD2L (Testis β-ACTIN).
SD23: for SD2M (CBB).

SD24-SD28: Single file for SD3.
SD24: for SD3F (Sperm AK1 and AK2).
SD25: for SD3G (Sperm β-ACTIN).
SD26: for SD3H (CBB).
SD27: for SD3J (Sperm ODF4).
SD28: for SD3K (Sperm β-TUBULIN).

SD29-SD34: Single file for SD4.
SD29: for SD4F (Sperm ODF2 and ODF1).
SD30: for SD4G (Sperm β-TUBULIN).
SD31: for SD4H (CBB).

SD32-SD34: Single file for SD5.
SD32: for SD5E (Sperm TEKTIN4).
SD33: for SD5F (Sperm β-ACTIN).
SD34: for SD5G (CBB).

SD35-SD36: Single file for SD6.
SD35: for SD6A (Sperm CATSPER3 and β-ACTIN).
SD36: for SD6E (CBB).

SD37-SD38: Single file for SD7.
SD37: for SD7E (Sperm AQP3).
SD38: for SD7F (CBB).

SD39-SD40: Single file for SD8.
SD39: for SD8E (Sperm AQP7).
SD40: for SD8F (CBB).

SD41-SD43: Single file for SD9.
SD41: for SD9B (Sperm AQP8).
SD42: for SD9E (CBB).
SD43: for SD9F (Sperm β-ACTIN).

SD44-SD46: Single file for SD10.
SD44: for SD10A (Sperm SEPTIN4 and SEPTIN7).
SD45: for SD10E (CBB).
SD46: for SD10F (Sperm β-TUBULIN).

SD47-SD49: Single file for SD11.
SD47: for SD11F (Sperm SLC22A14).
SD48: for SD11G (CBB).
SD49: for SD11H (Sperm β-TUBULIN).

SD50-SD51: Single file for SD12.
SD50: for SD12C (Sperm GAPDS).
SD51: for SD12F (CBB).

SD52-SD54: Single file for SD13.
SD52: for SD13A (Sperm GAPDH).
SD53: for SD13D (CBB).

SD54: Single file for SD14E IP.
SD54: for AK1 and AK2 of Tg and Odf4$^{-/-}$ (-/-), and the positive control AK1 and AK2).

SD55-SD56: Single file for SD15.
SD55: for SD15A for the rescued mice (RES).
SD56: for SD15D for the rescued mice (RES).

SD57-SD59: Single file for SD16.
SD57: for SD16E rescued study (RES).
SD58: for SD16F rescued study (RES).
SD59: for SD16G rescued study (RES).
